# TRPC6 specifically interacts with APP to inhibit its cleavage by γ-secretase and reduce Aβ production

**DOI:** 10.1038/ncomms9876

**Published:** 2015-11-19

**Authors:** Junfeng Wang, Rui Lu, Jian Yang, Hongyu Li, Zhuohao He, Naihe Jing, Xiaomin Wang, Yizheng Wang

**Affiliations:** 1 Laboratory of Neural Signal Transduction, Institute of Neuroscience, State Key Laboratory of Neuroscience, CAS Center for Excellence in Brain Science and Intelligence Technology, SIBS, CAS, 320 Yue-Yang Road, Shanghai 200031, China; 2 Graduate School of Chinese Academy of Sciences, University of Chinese Academy of Sciences, Shanghai 200031, China; 3 Department of Physiology and Neurobiology, Key Laboratory for Neurodegenerative Disorders of the Ministry of Education, Capital Medical University, and Beijing Institute for Brain Disorders, 10 Xitoutiao, You'anmen Wai, Beijing 100069, China; 4 Beijing Key Laboratory of Mental Disorders, Beijing Anding Hospital, Capital Medical University, Beijing 100088, China; 5 State Key Laboratory of Cell Biology, Institute of Biochemistry and Cell Biology, SIBS, Chinese Academy of Sciences, 320 Yue-Yang Road, Shanghai 200031, China

## Abstract

Generation of β-amyloid (Aβ) peptide in Alzheimer's disease involves cleavage of amyloid precursor protein (APP) by γ-secretase, a protease known to cleave several substrates, including Notch. Finding specific modulators for γ-secretase could be a potential avenue to treat the disease. Here, we report that transient receptor potential canonical (TRPC) 6 specifically interacts with APP leading to inhibition of its cleavage by γ-secretase and reduction in Aβ production. TRPC6 interacts with APP (C99), but not with Notch, and prevents C99 interaction with presenilin 1 (PS1). A fusion peptide derived from TRPC6 also reduces Aβ levels without effect on Notch cleavage. Crossing *APP/PS1* mice with *TRPC6* transgenic mice leads to a marked reduction in both plaque load and Aβ levels, and improvement in structural and behavioural impairment. Thus, TRPC6 specifically modulates γ-secretase cleavage of APP and preventing APP (C99) interaction with PS1 via TRPC6 could be a novel strategy to reduce Aβ formation.

## Abstract (web summary)

Attempts to treat Alzheimer's disease by targeting γ-secretase cleavage of APP into Aß have been unsuccessful, partially due to off-target effects. Here, the authors identify TRPC6 as a novel γ-secretase modulator, showing that it interacts with APP to regulate Aß levels while sparing Notch cleavage.

## Graphical abstract

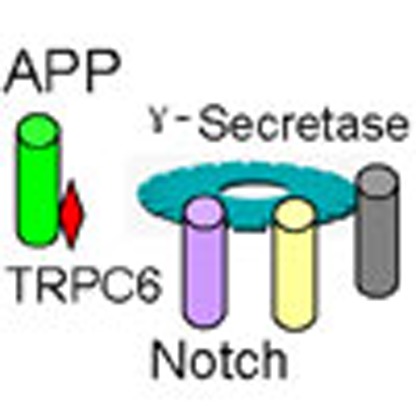

Alzheimer's disease (AD) is characterized by extracellular senile plaques and intracellular neurofibrillary tangles in autopsied brain tissues. Senile plaques are mainly composed of β-amyloid (Aβ) peptide, which is proposed to be responsible for AD pathogenesis^1^. The Aβ is generated through a sequential cleavage of amyloid precursor protein (APP) by β- and γ-secretases, while α-secretase cleavage precludes Aβ formation and produces neurotrophic sAPPα (ref. 2). To regulate APP cleavage by secretases and reduce Aβ production is a potential strategy for AD treatment.

The γ-secretase cleavage is the final step in Aβ production and attracts much attention in AD studies. However, γ-secretase has diverse substrates besides APP, such as Notch, E-/N-cadherin and ErbB-4 and γ-secretase cleavage of these proteins is essential for their physiological functions^3^,^4^. Drugs designed to inhibit γ-secretase activity may thus suppress the cleavage of a wide range of substrates concurrently, leading to many side effects. For example, administration of semagacestat, a potent γ-secretase inhibitor, resulted in reduced plasma Aβ levels, but worsened cognitive performance as well as enhanced skin cancer risk, immune system abnormalities and gastrointestinal symptoms, all of which were attributed to the inhibition of γ-secretase cleavage of Notch^5^. Indeed, semagacestat was found to be more potent to inhibit γ-secretase cleavage of Notch than that of APP^6^. Thus, specific modulation, instead of complete inhibition, of γ-secretase cleavage of APP might be an alternative avenue to reduce Aβ levels and treat the disease^7^.

The transient receptor potential canonical (TRPC) is a family of Ca^2+^-permeable nonselective cation channels, consisting of four subgroups, TRPC1, TRPC2, TRPC3/6/7 and TRPC4/5 (ref. 8). After activation by G-protein-coupled receptors or receptor tyrosine kinases, TRPC channels mediate Ca^2+^ influx and initiate cellular responses^9^. These channels have been reported to play important roles in development^10^ and diseases^11^,^12^. Recently, presenilin 2, a γ-secretase component, was reported to influence TRPC6 channel activity^13^, indicating that TRPC6 may be involved in Aβ production. Further, AD patients usually have severe synapse and neuron loss, leading to memory decline^14^,^15^,^16^, whereas TRPC6 promotes neuronal survival^12^,^17^, synapse formation^18^,^19^ and enhances spatial learning and memory^19^. We thus investigated whether TRPC6 affects Aβ production.

Here, we report that TRPC6 reduces Aβ levels both in cultures and in mice. TRPC6 interacts with APP (C99) to prevent the interaction between C99 and presenilin 1 (PS1) and thus suppresses γ-secretase cleavage of APP (C99) without affecting Notch cleavage. A fusion peptide derived from TRPC6 also reduces Aβ levels without effect on Notch cleavage. Therefore, targeting APP–PS1 interaction via TRPC6 may represent a novel intervention opportunity to reduce Aβ levels without side effects induced by inhibiting γ-secretase activity.

## Results

### TRPC6 regulated Aβ levels independent of its channel activity

**Figure 1:**
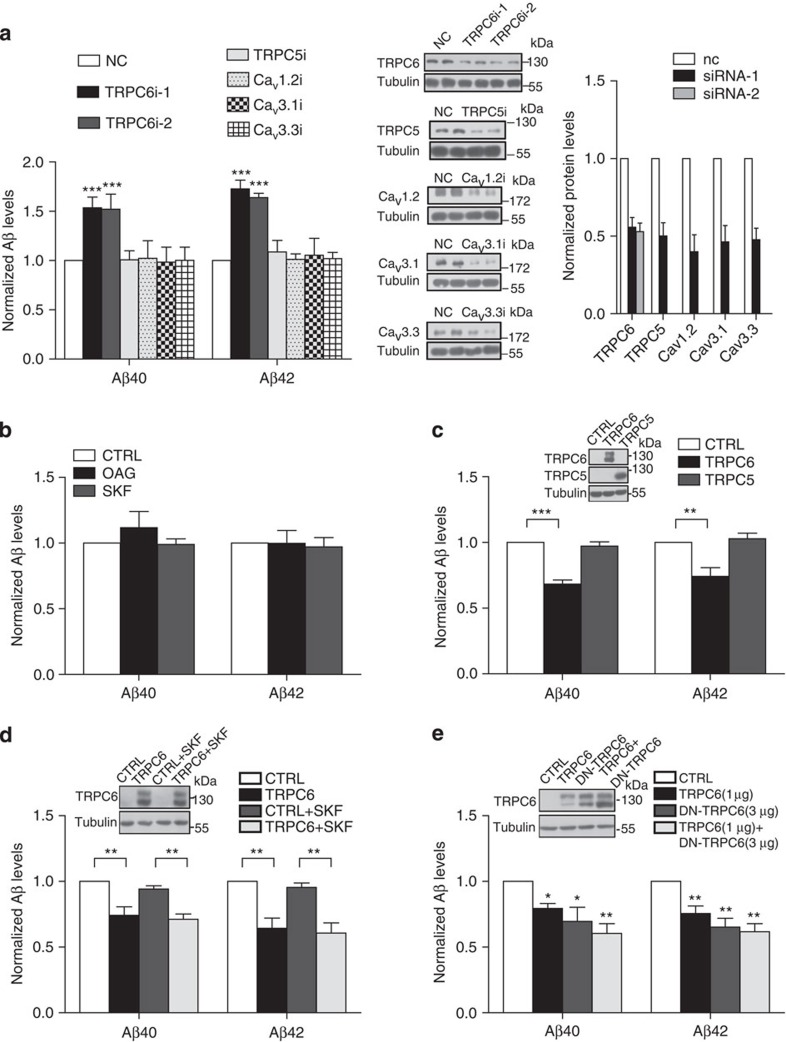
TRPC6 regulated Aβ levels in cultured cells. (**a**) Left: ELISA examination of Aβ levels in the medium of primary cultured cortical neurons transfected with the indicated siRNAs (*n*=3–4 in duplication). Immunoblot analysis (middle) and quantification (right) of the expression levels of the indicated proteins in the neurons transfected with siRNAs (*n*=3–4 in duplication). (**b**) Aβ levels in the medium of the cortical neurons treated with OAG (50 μM) or SKF96365 (2 μM) for 12 h (*n*=3). (**c**) Aβ levels in the medium of HEK293APP stable cells transfected with *TRPC5* or *6* for 2 days (*n*=5 in duplication). (**d**) Aβ levels in the medium of HEK293APP stable cells transfected with *YFP* or *TRPC6* for 1 day and then treated with vehicle or SKF96365 (1 μM) for 12 h. SKF96365 did not suppress TRPC6 inhibition of Aβ levels (*n*=4). (**e**) Aβ levels in the medium of HEK293APP cells transfected with indicated constructs for 2 days (*n*=4–5). Insets, representative immunobloting of indicated proteins. NC, negative control siRNA. CTRL, vehicle or transfection with *YFP*. Data were presented as means±s.e.m. of indicated numbers of independent experiments. One way ANOVA with Newman–Keuls *post hoc* test was performed. *P<0.05, **P<0.01, ***P<0.001 versus NC or CTRL.

We initially examined whether Ca^2+^ channels play a role in Aβ production because Ca^2+^ entry can affect α-secretase cleavage of APP^20^,^21^,^22^. We downregulated several Ca^2+^ channel proteins (Fig. 1a; Supplementary Fig. 10), including Ca_v_1.2, Ca_v_3.1 or Ca_v_3.3, L- or T-type voltage-dependent Ca^2+^ channel proteins; TRPC5 or TRPC6, nonselective cation channel proteins, in primary cultured rat cortical neurons and found that downregulating TRPC6, but not others, greatly enhanced both Aβ40 and Aβ42 levels determined by enzyme-linked immunosorbent assay (ELISA; Fig. 1a), suggesting that TRPC6 specifically regulates Aβ accumulation in cortical neurons. However, treatment of the neurons with OAG or SKF96365, agents known to activate or block TRPC channels^23^,^24^, respectively, did not influence the Aβ levels (Fig. 1b). These results suggest that TRPC6 regulates Aβ accumulation likely independent of its channel activity.

We then tested the effects of TRPC6 on Aβ levels in HEK293APP stable cells, a cell model commonly used for studying Aβ accumulation. All the Aβ40, Aβ42, Aβ38 and Aβ1-x levels in the culture medium of the cells transfected with *TRPC6* were markedly reduced (Fig. 1c; Supplementary Figs 1a and 10). In contrast, expressing *TRPC5* did not change the Aβ levels (Fig. 1c; Supplementary Fig. 10). These results show that TRPC6 specifically regulates Aβ accumulation in HEK293APP stable cells. Similar to its effect on Aβ accumulation in the neurons, SKF96365 did not suppress the inhibitory effect of TRPC6 on Aβ levels in HEK293APP cells (Fig. 1d; Supplementary Fig. 10). Meanwhile, expressing the dominant-negative form of TRPC6 (*DN-TRPC6*), a mutant known to suppress TRPC6 channel activity^25^, did not affect TRPC6 inhibition of Aβ accumulation (Fig. 1e; Supplementary Fig. 10). Moreover, expressing *DN-TRPC6* also reduced Aβ levels (Fig. 1e; Supplementary Fig. 10). All together, these results provide evidences that TRPC6 inhibition of Aβ accumulation is independent of its channel activity.

### TRPC6 prevented APP (C99) cleavage by γ-secretase

We next observed that expressing TRPC6 did not alter the APP levels, sAPPα production and expression of ADAM10 or BACE1 (Supplementary Fig. 2a,b; Supplementary Fig. 15). Further, the enzymatic activity of α- or β-secretase, determined by *in vitro* assay using their respective fluorogenic substrates was not changed by TRPC6 (Supplementary Fig. 2c). The Aβ can be cleared by enzymatic degradation or pumping into plasma^26^,^27^. However, the expression of insulin-degrading enzyme, neprilysin and clusterin, proteins known to affect Aβ clearance, was not changed by TRPC6 (Supplementary Fig. 2d,e; Supplementary Fig. 15). Furthermore, the Aβ levels in the medium and cell lysate of HEK293 cells assayed by the Aβ clearance test were not altered by TRPC6 (Supplementary Fig. 2f). Together, these results suggest that α- or β-secretase cleavage of APP and Aβ clearance are not affected by TRPC6.

**Figure 2:**
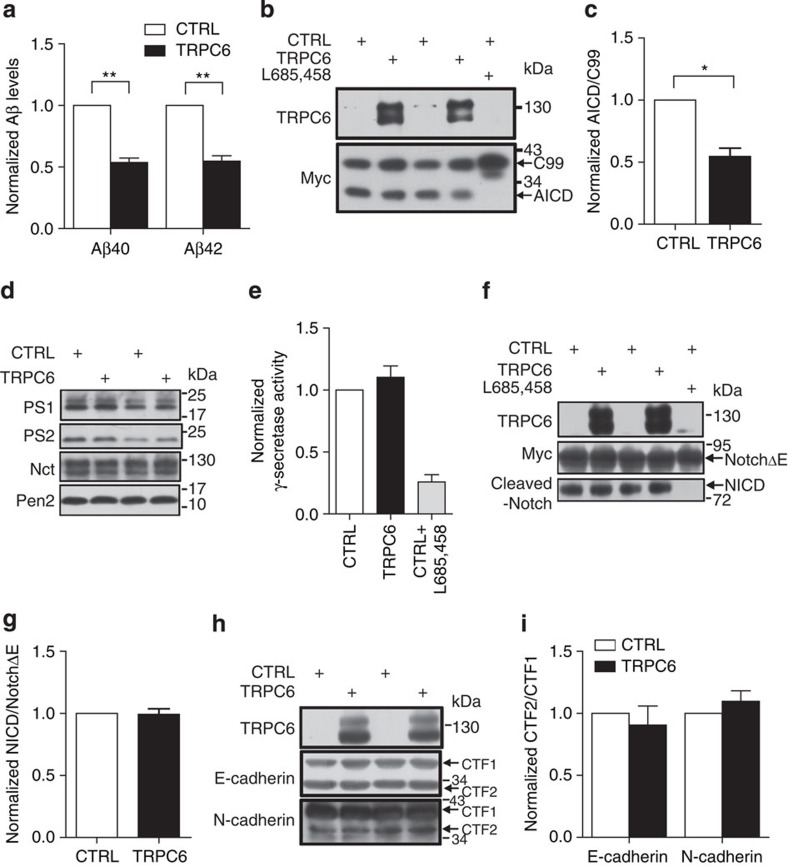
TRPC6 inhibited γ-secretase cleavage of APP (C99), but not that of other substrates. (**a**) Aβ levels in the medium of COS7C99 stable cells transfected with *YFP* or *TRPC6* for 2 days (*n*=3–4). (**b**) Immunoblot analysis of AICD in HEK293C99 cells transfected with *YFP* or *TRPC6* for 2 days. (**c**) Quantification of the ratio of AICD/C99 (*n*=3). (**d**) Immunoblots of presenilin 1 (PS1), PS2, nicastrin (Nct) and presenilin enhance 2 (Pen2) in HEK293APP cells transfected with *TRPC6* for 2 days. (**e**) γ-Secretase activity in the *in vitro* fluorogenic substrate assay of HEK293APP cells transfected with *TRPC6* for 2 days (*n*=3–5). (**f**) Immunoblot analysis of Notch intracellular domain (NICD) in HEK293APP cells transfected with *NotchΔE-myc* for 1 day and then further transfected with *YFP* or *TRPC6* for 2 days. (**g**) Quantification of the ratio of NICD/NotchΔE (*n*=4). (**h**) Immunoblots of CTF1 and CTF2 of E-/N-Cadherin in HEK293APP cells transfected with *YFP* or *TRPC6* for 2 days. (**i**) Quantification of CTF2/CTF1 of E-/N-Cadherin (*n*=3–4). CTRL, transfection with *YFP* or pcDNA3.1. L658,458: γ-secretase inhibitor. Data were presented as means±s.e.m. of indicated numbers of independent experiments. Two-tailed Student's *t*-test was performed. *P<0.05, **P<0.01 versus CTRL.

We then examined whether γ**-**secretase cleavage of APP was affected by TRPC6. Cleavage of C99 by γ**-**secretase releases Aβ peptides and APP intracellular domain (AICD)^28^. TRPC6 greatly reduced both Aβ40 and Aβ42 levels in the culture medium of COS7 cells stably expressing C99 (Fig. 2a). TRPC6 also downregulated AICD generation in HEK293C99 stable cells (Supplementary Figs 2g, 11 and 15; Fig. 2b,c). In addition, TRPC6 did not affect CTF (C-terminal fragments of APP) levels either in HEK293APP cells (Supplementary Fig. 2h,I; Supplementary Fig. 15) or in *APP/PS1* mice (Supplementary Fig. 2j,k; Supplementary Fig. 15). Further, expression of PS1, PS2, nicastrin or presenilin enhancer 2, proteins known as the γ-secretase components, was not changed by TRPC6 (Fig. 2d; Supplementary Figs 2l and 11). Consistently, the γ-secretase activity determined by *in vitro* assay using the fluorogenic substrate was unchanged by TRPC6 (Fig. 2e). We also examined whether TRPC6 affected γ-secretase cleavage of Notch by detecting Notch intracellular domain level generated from NotchΔE, an immediate substrate of γ**-**secretase^29^. As shown in Fig. 2f,g and Supplementary Fig. 11, TRPC6 did not alter the Notch intracellular domain level, suggesting that TRPC6 did not inhibit γ-secretase cleavage of Notch. We further examined the CTF1 and CTF2 levels of E-/N-Cadherin, known as the endogenous substrates of γ-secretase. As shown in Fig. 2h,i and Supplementary Fig. 11, TRPC6 did not alter the CTF2/CTF1 ratio of E-/N-Cadherin, suggesting that TRPC6 did not inhibit γ-secretase cleavage of E-/N-Cadherin. Together, these results suggest that TRPC6 specifically reduces γ**-**secretase cleavage of C99, but does not act as a general γ**-**secretase inhibitor.

**Figure 3:**
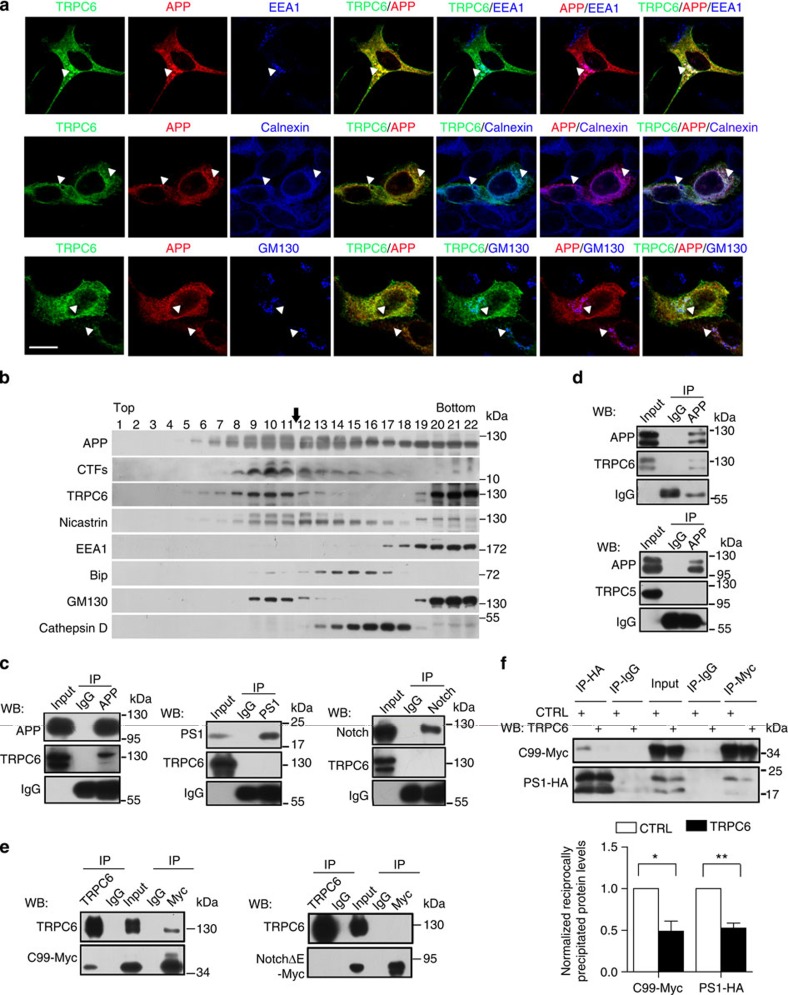
TRPC6 interacted with APP (C99). (**a**) Immunocytochemical analysis of HEK293 cells transfected with *APP-Myc* and *TRPC6-HA* for 3 days. EEA1, Calnexin and GM130 are markers for early endosome, ER and Golgi, respectively. Arrow heads indicated the co-localized signals. Scale bar, 20 μm. (**b**) Immunoblots of indicated proteins after fractionation of mouse brain lysates. Bip and Cathepsin D are markers for ER and lysosome, respectively. Arrow indicated alignment of two gels with 11 lanes running in parallel. (**c**) Wild-type mouse brain lysates precipitated with the antibody against APP, PS1 or Notch, and immunoblotted with indicated antibodies. (**d**) Lysates of HEK293APP stable cells transfected with *TRPC5* or *6* for 2 days precipitated with APP antibody, and immunoblotted with indicated antibodies. (**e**) Lysates of HEK293TRPC6 stable cells transfected with *C99-Myc* or *NotchΔE-Myc* for 1 day, treated with 10 μM L685,458 for 12 h and then precipitated with Myc or TRPC6 antibody, and immunoblotted with indicated antibodies. (**f**) Lysates of HEK293C99-Myc stable cells transfected with *PS1-HA* together with *YFP* or *TRPC6* for 2 days and precipitated with Myc or HA antibody, and immunoblotted with indicated antibodies. Lower, quantification of the reciprocal precipitated protein levels of C99 and PS1 (*n*=4). CTRL, transfection with YFP. Data were presented as means±s.e.m. of indicated numbers of independent experiments. Two-tailed Student's *t*-test was performed. *P<0.05, **P<0.01 versus CTRL.

Certain proteins can interact with APP (C99) to regulate Aβ production^30^. We next studied whether TRPC6 and APP (C99) were subcellularly co-localized. As the anti-TRPC6 antibody on hand cannot be used for immunostaining, we transfected HEK293 cell with *TRPC6-HA* and *APP-Myc* (*C99-Myc*) and stained with antibodies against HA- and Myc-tag. We found that TRPC6 and APP (C99) might co-localize in certain organelles (Fig. 3a; Supplementary Fig. 3a). These results provide the anatomic evidence to support the possible interaction between TRPC6 and APP. Next, in the fractionation experiments with mouse brain lysates, endogenous TRPC6 and APP (CTF) were also found in the same fractions (Fig. 3b; Supplementary Fig. 12). We then explored whether TRPC6 and APP were in the same complexes. Immunoprecipitation of mouse brain lysates, HEK293 cell or cortical neuronal lysates by APP antibody revealed that TRPC6 was in the precipitated complexes (Fig. 3c; Supplementary Figs 3b,c, 13 and 16). In contrast, TRPC6 was not found in the complexes precipitated by PS1, Notch or APLP2 antibody (Fig. 3c; Supplementary Figs 3b–f, 13, 16 and 17), suggesting that TRPC6 selectively interacted with APP, but not with PS1, Notch or APLP2. Similarly, TRPC6 was found in the complexes precipitated by APP antibody in HEK293APP cells overexpressing TRPC6 (Fig. 3d; Supplementary Fig. 13), and the precipitated TRPC6 level was increased when its expression was enhanced (Supplementary Fig. 4a,b; Supplementary Fig. 17). In addition, TRPC6 was found in the complexes precipitated by APP antibody in HEK293TRPC6 cells overexpressing APP-I716F or APP-V717F, two familial AD-linked APP variants around γ-secretase cleavage sites (Supplementary Figs 4c–f and 17). In contrast, TRPC5 was not precipitated by APP antibody in HEK293APP cells overexpressing TRPC5 (Fig. 3d; Supplementary Fig. 13). Moreover, TRPC6, but not TRPC5, and C99 were found in the complexes by reciprocal precipitation experiments in HEK293C99 cells (Supplementary Figs 4g and 17). In addition, NotchΔE was not found in the complexes precipitated by TRPC6 antibody (Fig. 3e; Supplementary Fig. 14). All together, these results point to a possibility that TRPC6 selectively interacts with APP (C99) to specifically suppress its cleavage by γ-secretase.

We next explored whether TRPC6 affected the interaction between C99 and PS1. The C99-Myc and PS1-HA in HEK293C99 cells were found in the complexes precipitated with the antibody against HA- or Myc-tag reciprocally (Fig. 3f; Supplementary Fig. 14). However, the reciprocal precipitation of C99 and PS1 was markedly inhibited when TRPC6 was expressed (Fig. 3f; Supplementary Fig. 14). It has been reported that PS2 can affect TRPC6-mediated Ca^2+^ entry in HEK293 cells^13^. We next explored whether APP could regulate the activity of TRPC6. Application of hyperforin, an agonist of TRPC6 (refs 18, 31), induced Ca^2+^ influx in HEK293TRPC6 cells and this Ca^2+^ entry was not affected by APP (Supplementary Fig. 4h,i). Together, these results suggest that TRPC6 prevents APP (C99) interaction with PS1 and APP does not affect the channel activity of TRPC6.

### TM2 domain was essential for TRPC6 to reduce Aβ

**Figure 4:**
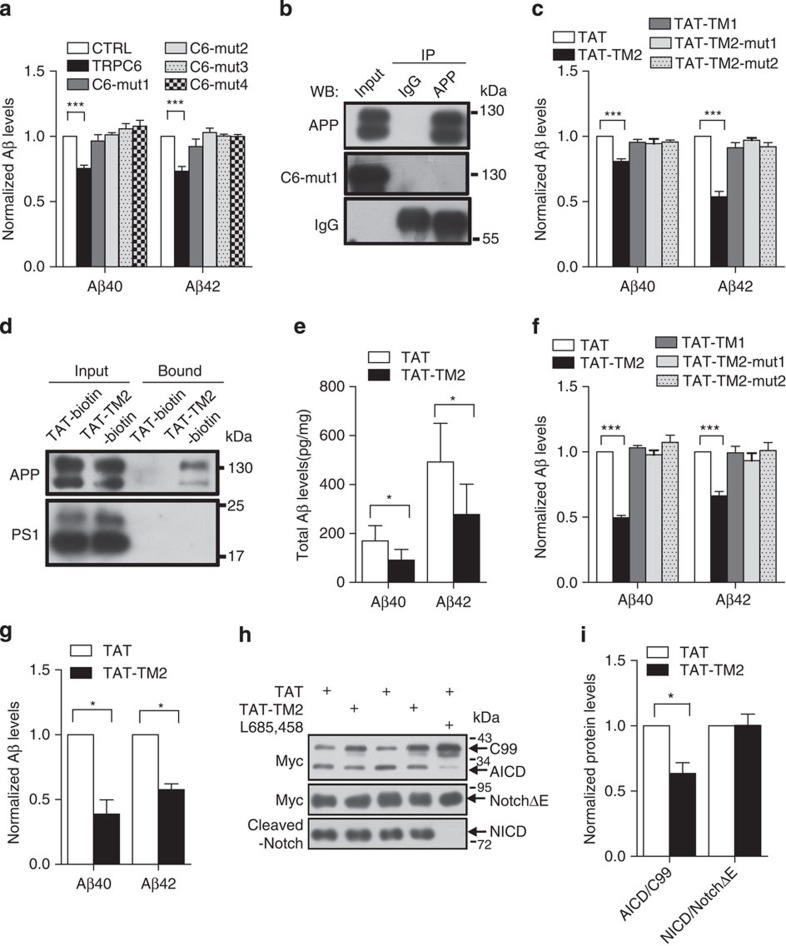
TAT-TM2 reduced Aβ levels *in vitro* and *in vivo*. (**a**) ELISA examination of Aβ levels in the medium of HEK293APP cells transfected with *TRPC6* or its mutants within second transmembrane domain (TM2) for 2 days (*n*=3–6). (**b**) Lysates of HEK293APP stable cells transfected with *C6-mut1* for 2 days precipitated with APP antibody and immunoblotted with indicated antibodies. (**c**) The Aβ levels in the medium of HEK293APP cells treated with 2 μM indicated peptides for 12 h (*n*=3–6). (**d**) Lysates of HEK293APP stable cells incubated with 5 μM TAT-biotin or TAT-TM2-bitoin for 6 h precipitated with avidin beads and immunoblotted with indicated antibodies. (**e**) ELISA examination in a blinded manner of Aβ levels in the brain lysates of 5 month male *APP/PS1* mice intra-peritoneally injected with 200 μl 2.5 mM TAT or TAT-TM2 for 3 h (*n*=8 mice). (**f**) The Aβ levels generated in the *in vitro* purified C99 cleavage assay treated with 2 μM of indicated peptides for 2 h (*n*=4). (**g**) Aβ levels in the medium of COS7C99 stable cells treated with 5 μM TAT-TM2 for 12 h (*n*=3–4). (**h**) Immunoblots of AICD in HEK293C99 cells treated with 5 μM TAT-TM2 for 12 h or NICD in HEK293APP cells transfected with *Notch≜E-myc* for 1 day and then treated with 5 μM TAT-TM2 for 12 h. (**i**) Quantification of the ratio of AICD/C99 or NICD/NotchΔE (*n*=3–4). CTRL, transfection with *YFP*. Data were presented as means±s.e.m. of indicated numbers of independent experiments. Two-tailed Student's *t*-test was performed for two groups, and one way ANOVA with Newman–Keuls *post hoc* test was performed for more than two groups. *P<0.05, ***P<0.001 versus CTRL or TAT.

To provide direct evidence that TRPC6 interaction with APP indeed inhibited APP cleavage by γ-secretase and Aβ formation, we investigated which domain of TRPC6 mediated its inhibitory effect on Aβ production. TRPC6 has six transmembrane domains and cytoplasmic N- and C-terminals (Supplementary Fig. 5a). As the cytoplasmic tail of C99 was reported to interact with several cytosolic proteins and these interactions may regulate Aβ production^30^, we first hypothesized that cytoplasmic terminals of TRPC6 might interact with the cytoplasmic domain of C99 to reduce Aβ production. We transfected HEK293APP cells with the N or C terminus (1–405 or 725–930 aa (amino acids)) of *TRPC6* and examined Aβ levels. These constructs did not affect Aβ generation (Supplementary Fig. 5b). After a series of truncation experiments (Supplementary Fig. 5c), we found that all the constructs containing 437–508 aa of TRPC6 reduced Aβ levels, whereas the constructs without this domain did not (Supplementary Fig. 5d), suggesting that 437–508 aa of TRPC6 was important for its inhibition of Aβ production. This domain contains the first and second transmembrane regions and the first extracellular loop. When the second transmembrane (TM2) region was mutated, by point mutation (*C6-mut1* and *C6-mut2*, Supplementary Fig. 5e), replacement (*C6-mut3*) or reversal (*C6-mut4*), TRPC6 was not able to reduce Aβ levels (Fig. 4a), indicating that the TM2 domain was essential for TRPC6 to regulate Aβ generation. Immunoprecipitation experiment revealed that C6-mut1 was not in the complexes precipitated by APP antibody (Fig. 4b; Supplementary Fig. 14), suggesting that TRPC6 may interact with APP through TM2 domain to inhibit Aβ formation. To rule out the possibility that mutation of TRPC6 might affect its subcellular localization, we transfected HEK293 cell with *C6-mut1-HA* (Supplementary Fig. 5e) and *APP-Myc*, and stained with HA and Myc antibodies. The C6-mut1 and APP might co-localize in certain organelles (Supplementary Fig. 5f), similar to wild-type (WT) TRPC6 and APP (Fig. 3a).

### TAT-TM2 inhibited Aβ accumulation *in vitro* and *in vivo*

To mimic TRPC6 inhibition of Aβ formation, a cell-permeable peptide TAT-TM2 containing the 487–508 aa of TRPC6 was synthesized and TAT-TM2, but not its mutated form (TAT-TM2-mut1, 2, Supplementary Fig. 6a) or TAT-TM1, greatly suppressed Aβ production in HEK293APP cells (Fig. 4c). Further, TAT-TM2 dose-dependently reduced Aβ levels in HEK293APP cells and in the primary cultured cortical neurons (Supplementary Fig. 6b,c). To determine the amino acids important for the inhibitory effect, TAT-TM2-N (N-terminal 10 aa of TM2 fused to TAT) and TAT-TM2-C (C-terminal 11 aa of TM2 fused to TAT) were synthesized. The TAT-TM2-C inhibited Aβ generation, whereas TAT-TM2-N did not (Supplementary Fig. 6d), suggesting that the C-terminal 11 aa of TM2 was critical for reducing Aβ formation. Indeed, mutations within this domain blocked the effect of TAT-TM2-C on reducing Aβ levels (Supplementary Fig. 6e). In the experiment to test whether TAT-TM2 also binds to APP, we found that TAT-TM2-biotin, but not TAT-biotin, interacted with APP similar to the full-length TRPC6 (Fig. 4d and Supplementary Fig. 14), whereas it did not bind to TRPC6 (Supplementary Figs 7a and 18). Addition of TAT-TM2 prevented the interaction between APP and TAT-TM2-biotin or TRPC6 (Supplementary Figs 7b–e and 18). Further, assayed by a double-blinded manner, injection of TAT-TM2 into *APP/PS1* mice led to a marked reduction in Aβ levels in the mouse brain (Fig. 4e). All these results show that TAT-TM2 reduces Aβ accumulation in cultures and in mice.

To show whether TAT-TM2 can prevent γ-secretase cleavage of APP (C99), we carried out the *in vitro* purified C99 cleavage assay. TAT-TM2, but not its mutated forms (TAT-TM2-mut1, 2) or TAT-TM1, inhibited Aβ generation (Fig. 4f). Further, TAT-TM2 dose-dependently reduced Aβ levels in the *in vitro* C99 cleavage assay (Supplementary Figs 6f, 7f,g and 18). The Aβ production in COS7 cells stably expressing C99 and the AICD generation from HEK293C99 cell, all were suppressed when TAT-TM2 was applied (Fig. 4g–i; Supplementary Fig. 14), suggesting that TAT-TM2 prevented γ-secretase cleavage of APP (C99). However, TAT-TM2 did not affect Notch cleavage by γ-secretase (Fig. 4h,i; Supplementary Fig. 14). Together, these results suggest that TAT-TM2 reduces Aβ levels without effect on γ-secretase cleavage of Notch.

### TRPC6 reduced Aβ to improve spatial learning of AD mice

**Figure 5:**
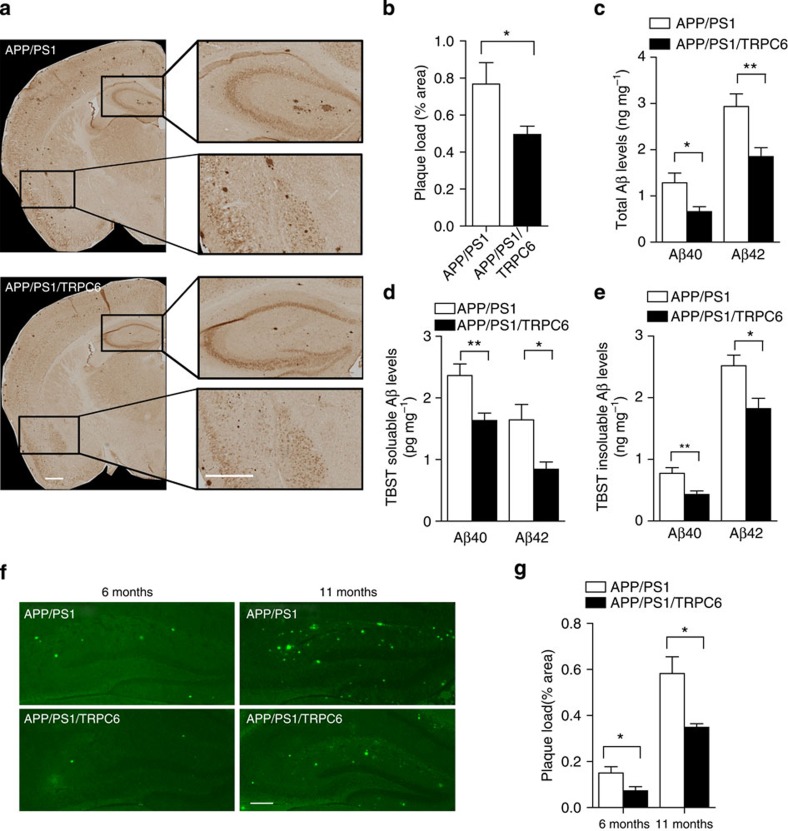
The Aβ levels were decreased in *APP/PS1/TRPC6* mice. (**a**) Representative images of amyloid plaque immunostained with Aβ antibody (6E10) on brain sections from 6 months female *APP/PS1* and *APP/PS1/TRPC6* mice. Scale bar, 500 μm. (**b**) Quantification of the plaque load in indicated mouse brain sections shown in **a** (*n*=8). ELISA of total (**c**) TBST soluble (**d**) or insoluble (**e**) Aβ levels in the forebrain lysates of 6 month female indicated mice (*n*=8). Representative images (**f**) and quantification (**g**) of thioflavin S staining of amyloid plaque in the hippocampal region of 6 and 11 months *APP/PS1* and *APP/PS1/TRPC6* mice with B6C3 background (*n*=4–5 mice). Scale bar, 200 μm. Data were presented as means±s.e.m. Two-tailed Student's *t*-test was performed. *P<0.05, **P<0.01 versus *APP/PS1*.

To test whether TRPC6 could regulate Aβ levels in animals, we crossed *TRPC6* transgenic mice, in which *TRPC6* was overexpressed in the forebrain excitatory neurons^19^, with *APP/PS1* mice, a mouse model commonly used to study Aβ formation^32^. Amyloid plaques were examined by Aβ immunostaining with 6E10 antibody on the brain sections of *APP/PS1* and *APP/PS1/TRPC6* mice (Fig. 5a; Supplementary Fig. 8a). The plaque load was markedly reduced in *APP/PS1/TRPC6* male (Supplementary Fig. 8b) and female (Fig. 5b) mice compared with that in *APP/PS1* mice. Plaque staining with Aβ specific antibody MOAB-2 (ref. 33) confirmed that the plaque load was greatly reduced in *APP/PS1/TRPC6* mice compared with that in *APP/PS1* mice (Supplementary Fig. 8f–i). Total Aβ levels in guanidine-HCl (GuHCl) extraction of *APP/PS1/TRPC6* male (Supplementary Fig. 8c) and female (Fig. 5c) mouse forebrains were greatly reduced than those of *APP/PS1* mouse forebrains. The TBST soluble and insoluble Aβ levels in *APP/PS1/TRPC6* male (Supplementary Fig. 8d,e) and female (Fig. 5d,e) mice were also substantially reduced compared with those in *APP/PS1* mice. To further confirm the effect of TRPC6, we examined whether TRPC6 could reduce amyloid plaque in *APP/PS1* mice with another genetic background. The plaque load in the hippocampus of *APP/PS1/TRPC6* mice in B6C3 background stained with thioflavin S (Fig. 5f) was also greatly reduced than that in *APP/PS1* mice (Fig. 5g). Taken together, TRPC6 reduces Aβ accumulation in *APP/PS1* mice.

**Figure 6:**
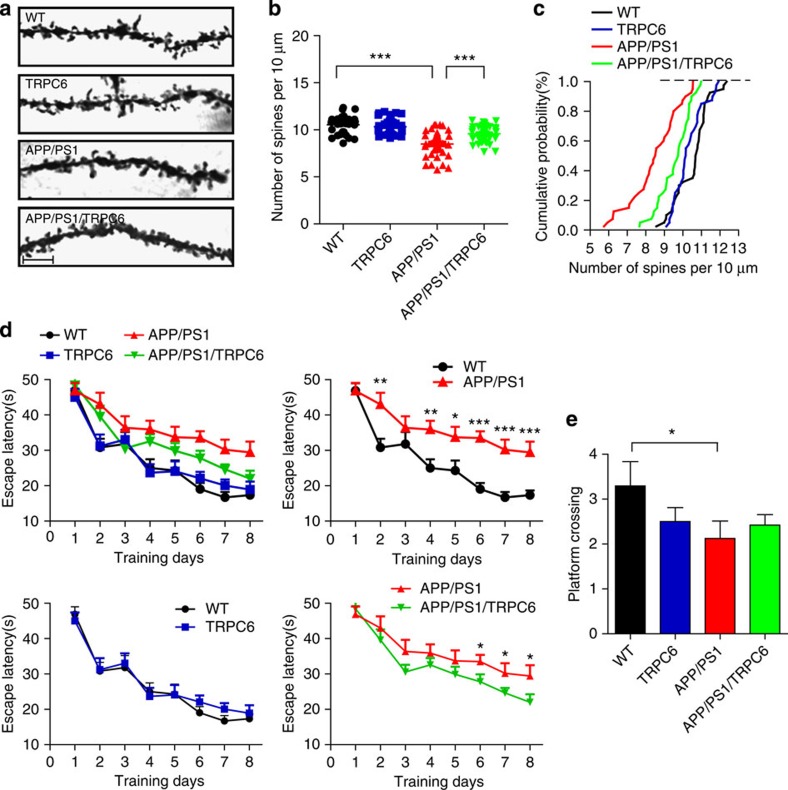
*APP/PS1/TRPC6* mice showed improvement in spatial learning ability at 11 months. (**a**) Representative images of Golgi staining of hippocampal CA1 neurons in the four groups of mice at 11 month. Scale bar, 5 μm. (**b**) Quantification of spine number per 10 μm of dendrite (*n*=40 neurons from 3–5 mice). (**c**) Cumulative probability analysis of the spine density shown in **b**. (**d**) Escape latency of the four groups of mice at 11 months in the training session of Morris water maze (*n*=15–19). (**e**) Platform crossing number of the mice at 11 month in the probe session of Morris water maze (*n*=14–19). Data were presented as means±s.e.m. One way ANOVA with Newman–Keuls *post hoc* test was performed for spine quantification and repeated measures and multivariate analysis in general linear model with LSD *post hoc* test was performed for behavioural study. *P<0.05, **P<0.01, ***P<0.001 versus WT or *APP/PS1*.

As synapse loss was found to be best correlated with dementia degree^14^,^15^ and Aβ was proposed to be responsible for the synapse loss and dementia, we next asked whether reduced Aβ levels by TRPC6 could improve AD pathology. At 6 months, the spine density, an indicator to evaluate synapse numbers revealed by Golgi staining, of *TRPC6* mouse hippocampus was higher than that of WT mice (Supplementary Fig. 9a–c). But at 11 month the spine density between *TRPC6* mice and WT mice was likely similar (Fig. 6a–c). The spine density of *APP/PS1* mice either at 6 or 11 months was greatly reduced than that of WT mice, whereas it was markedly increased in *APP/PS1/TRPC6* mice than that in *APP/PS1* mice, suggesting that TRPC6 prevented the spine loss in *APP/PS1* mice. In the behavioural test, we found that at 6 months, *TRPC6* mice performed better than WT mice both in the training and probe session of Morris Water Maze (Supplementary Fig. 9d,e), while at 11 months, there was no difference in the performance between *TRPC6* and WT mice (Fig. 6d,e). However, compared with *APP/PS1* mice, *APP/PS1/TRPC6* mice spent shorter time to find the hidden platform in training session at 11 months (Fig. 6d), and crossed the platform region more times in the probe test at 6 months (Supplementary Fig. 9e). Therefore, overexpression of *TRPC6* in neurons improves learning and memory in *APP/PS1* mice. All these results are consistent with an explanation that TRPC6 downregulates Aβ levels to maintain the structural and behavioural plasticity of AD mouse brains.

## Discussion

In this study we reported that TRPC6 could function as a novel endogenous γ-secretase modulator and specifically regulate Aβ production. Several lines of evidence support this conclusion. First, TRPC6 inhibited γ-secretase cleavage of APP without effect on α- or β-secretase cleavage of APP, Aβ clearance or γ-secretase cleavage of other substrates tested (Fig. 2; Supplementary Figs 2, 11 and 15). Second, TRPC6 selectively interacted with APP (C99), but not with Notch (Fig. 3c,e; Supplementary Figs 3b,c,13,14 and 16). Third, the fusion peptide TAT-TM2 prevented γ-secretase cleavage of APP, but not that of Notch (Fig. 4h,I; Supplementary Fig. 14). Finally, elevating TRPC6 in the forebrain neurons reduced Aβ levels and improved structural and behavioural plasticity of *APP/PS1* mice (Figs 5, 6; Supplementary Figs 8 and 9). Thus, affecting TRPC6 levels may be a new strategy to specifically inhibit γ-secretase cleavage of APP and reduce Aβ production.

Several compounds have been developed to target γ-secretase, but they were not successful in clinical trials to treat AD patients^5^,^34^. Although different reasons may have contributed to the failures, the unwanted side effects due to the strong inhibition of γ-secretase by the compounds prevent clinical use of these agents. Therefore, the search for a specific modulation of γ-secretase cleavage of APP without affecting other substrates, including Notch^7^, is urgently needed. Several proteins, including GPR3 (ref. 35), Aph1B/C^36^, GSK3α (ref. 37), CK1 (ref. 38) and DOR^39^, have been reported to specifically enhance γ-secretase cleavage of APP to promote Aβ generation. However, proteins which can specifically downregulate Aβ production are less reported. It has been shown that downregulation of TMP21, reported as a γ-secretase component, increases Aβ levels without effect on cleavage of Notch^40^. However, elevating TMP21 expression in cells could not reduce Aβ levels^40^, and overexpressing TMP21 in mice causes post-natal growth retardation, severe neurological problems and premature death^41^. Here, we report that TRPC6 interaction with APP leads to the specific reduction in Aβ production and improvement in structural and behavioural plasticity in *APP/PS1/TRPC6* mice. It is thus likely that the substrate selectivity of γ-secretase could be finely regulated by additional proteins, such as TRPC6, to confer the selective modulation of Aβ production.

While a few proteins have been found to regulate the substrate selectivity of γ-secretase, the mechanism remains largely unknown. Recently, γ-secretase-activating protein (GSAP) was reported to increase γ-secretase cleavage of APP (C99) and Aβ generation, whereas Notch cleavage was not influenced^42^. It has been shown that GSAP could specifically interact with C99, but not with Notch, an observation similar to TRPC6 reported here (Fig. 3c,e; Supplementary Figs 3b,c, 13, 14 and 16). Thus, selective substrate targeting by interaction may confer a specific modulation of γ-secretase cleavage. Actually, it has been reported that small-molecule γ-secretase modulators, which regulate γ-secretase cleavage of APP, but not that of Notch, bind to C99 with a high affinity, but to Notch with a much lower affinity^43^. However, different from GSAP, which also binds to PS1, TRPC6 does not interact with PS1. Several other proteins have been reported to interact with PS1 to modulate γ-secretase cleavage of APP^36^,^39^,^40^. In this context, the APP-specific interaction of TRPC6 to regulate Aβ generation may basically minimize the risk of inhibiting γ-secretase to avoid unexpected side effects, a critical problem encountered in clinical trials.

The TRPC channels are nonselective cation channels which play important roles in development^10^ and diseases^11^,^12^. To date all the reported functions of TRPC6 channels require its channel activities. Here, we reported that TRPC6 inhibited γ-secretase cleavage of APP likely through protein–protein interaction, but not requiring its channel activity. Actually, Ca_v_1.2, an L-type Ca^2+^ channel protein, has been reported to regulate transcription of certain genes independent of its channel activity, but through the C-terminal fragment^44^. TRPM6 and TRPM7, two channel proteins with kinase activity, have been found to phosphorylate myosin IIA and annexin I through their kinase domains^45^. In addition, TRPC1 could promote neurite outgrowth independent of its channel activity^46^. Thus, TRPC6 is a new channel protein to exert cellular functions independent of its channel activity. It is not clear for now whether TRPC6 interaction with APP is regulated. Since downregulation of TRPC6 and DN-TRPC6, all affect Aβ production, the level of TRPC6 is critical for its modulation of Aβ production. Therefore, it will be informative to examine the expression levels of TRPC6 in AD patients.

In conclusion, our results showed that TRPC6 regulated Aβ levels independent of its channel activity. TRPC6 inhibited γ-secretase cleavage of APP, but not that of Notch, through its selective interaction with APP. These results suggest that preventing APP interaction with PS1 via TRPC6 could be a novel strategy to reduce Aβ formation and reveal a novel cellular function of TRPC6. Finding molecule(s) functioning as TAT-TM2, which is able to reduce Aβ production without affecting Notch cleavage, may provide novel intervention candidates to reduce Aβ levels and treat AD patients while bypassing the side effects induced by the direct inhibition of γ-secretase activity.

## Methods

### Animals

All animal experiments were performed in accordance with the Institutional Animal Care and Use Committee of the Institute of Neuroscience, Shanghai, China. *APP/PS1* mice were from Jackson laboratory (#004462) in B6C3 background, and *TRPC6* transgenic mice were constructed in C57 background^19^. Animal age and sex is provided in the related figure legends. To obtain mice with the same genetic background, *APP/PS1* mice or *TRPC6* mice were first crossed with C57 mice for several generations or with C3H mice, respectively. Then, *APP/PS1* mice and *TRPC6* mice in the same background were crossed and off springs were used. All the mice were randomized according to their genotype.

### Thioflavin S staining, immunohistochemistry and immunocytochemistry

Briefly, mouse brain sections were stained with Thioflavin S (Sigma, 20 μg ml^−1^) and differentiated in 70% ethanol. Immunostaining of amyloid plaque with Aβ antibody was performed according to the instruction of VectaStain Universal ABC kit (VECTOR, PK-6200). Briefly, mouse brain sections were first quenched with H_2_O_2_ and antigen retrieved in formic acid. After incubation with 6E10 or MOAB-2 antibody overnight, sections were further incubated with secondary antibody (VECTOR, PK-6200, 1:50), ABC complex and visualized with DAB (VECTOR, SK-4100) as a substrate. These sections were examined under Neurolucidar (Nikon) after mounting. For immunocytochemistry, HEK293 cells from ATCC were co-transfected with *TRPC6-HA/APP-Myc*, *TRPC6-HA/C99-Myc* or *C6-mut1-HA/APP-Myc*. The cells were trypsinized 1 day after transfection and cultured on 12 mm coverslips for 2 days. The cells were then fixed with paraformaldehyde (PFA), permeabilized with Triton, blocked with normal donkey serum and then stained with primary antibodies overnight, and subsequently incubated with Alexa Flour 488/546/647–conjugated secondary antibodies (Invitrogen and Jackson, 1:1,000). Images were obtained using FV10I confocal microscope (Olympus).

### Aβ ELISA

Mouse forebrains were dissociated and homogenized with automatic homogenizer (Shanghai Jingxin, JXFSTPRP-48) in TBST. The homogenate was mixed with GuHCl and rotated at room temperature for total Aβ quantification. For TBST soluble and insoluble Aβ quantification, the homogenate was centrifuged and the supernatant was collected for TBST soluble Aβ, and the pellet was further homogenized in GuHCl for TBST insoluble Aβ. Aβ levels of the mouse forebrain and in culture medium of the cells were quantified by the ELISA kit (Invitrogen, KHB3482 and KHB3544, or IBL, 27717 and 27729).

### Aβ clearance assay

Aβ clearance assay was carried out as previously described^47^. HEK293 cell was transfected with *YFP* or *TRPC6* for 1 day, and switched to serum-free DMEM with 1 μg ml^−1^ dissolved Aβ42 (American Peptide) and 10 μg ml^−1^ BSA for 12 h. After incubation, the culture medium was collected and Aβ42 level was measured by ELISA. The cells were washed with PBS and lysed in 100 μl of 5 M GuHCl, and Aβ42 level was measured by ELISA.

### Golgi staining

Golgi staining was carried out using Rapid GolgiStain Kit (FD Neurotechnologies, PK401) according to the instruction of the manufacturer. Briefly, after immersion in the impregnation solution for 1 week, mouse brains were cut into 100 μM sections. Sections were stained and dehydrated in graded ethanol, cleared in xylene and mounted. The sections were examined with Zeiss LSM 510 confocal microscope.

### Morris water maze

Morris water maze experiment was conducted as previously described^19^. The water maze was a circular pool of 1 m in diameter and divided into four equal quadrants with a platform submerged 1 cm underneath the water surface and kept in the centre of a certain quadrant. During training session, animals were placed into water in randomized order of quadrant each day, with every quadrant once per day. Training session consisted of 8 days, four trials per day, and the time spent to find the hidden platform was recorded as escape latency for each mice. After training, a probe test was carried out with removed platform. Mice were allowed to swim for 1 min and the number of platform area crossing was recorded. Animals showed obvious behavioural abnormality, like never stand on the platform, always rotate or swim around the edge of the maze, were excluded for analysis. Behavioural data were statistically analysed using repeated measures and multivariate analysis in general linear model with LSD *post hoc* test (SPSS 16.0).

### Immunoprecipitation

For the immunoprecipitation, mouse brains or cells were lysed in the buffer containing 0.01 M PB (pH 7.4), 1% NP40, 1% DOC, 0.1% SDS, 0.15 M NaCl and 2 mM EDTA or 20 mM Tris-HCl (pH 8), 137 mM NaCl, 10% glycerol, 1% NP40 and 2 mM EDTA, respectively. The antibodies or control IgG and protein A/G beads were sequentially added into the precipitation system and rotated at 4 °C overnight. The beads were washed several times and the immunoprecipitated proteins were resolved by SDS–polyacrylamide gel electrophoresis and analysed by immunoblots.

### *In vitro* secretase activity assay

Briefly, cells were first homogenized and centrifuged, and secretases in the pellet were re-solublized. The α-secretase activity was assayed in 100 mM sodium acetate (pH 7.0) with 2 μg fluorogenic substrate (Calbiochem, 565767), while β-secretase activity assayed in 100 mM sodium acetate (pH 4.5) with 2 μg fluorogenic substrate (Calbiochem, 565758) and γ-secretase activity assayed in 50 mM Tris-HCl (pH 6.8), 2 mM EDTA and 0.25% CHAPSO (w/v) with 3 μg fluorogenic substrate (Calbiochem, 565764). After incubation at 37 °C, the fluorescence intensities were measured with an excitation wavelength at 340 nm and an emission wavelength at 490 nm for α-secretase, 350 and 490 nm for β-secretase, 355 and 440 nm for γ-secretase.

### Cell cultures and transfection

HEK293APP stable cell line (kindly provided by Dr Haiyan Zhang) was maintained in DMEM with 10% FBS and COS7C99 stable cell line (kindly provided by Dr Dai Zhang) in MEM/F12 with 5% FBS supplemented with 1% NEAA and 200 mM Glutamax. Transfection was done using Lipofectamin 2000 (Invitrogen, 11668-019). Primary rat cortical neurons were dissected from embryonic day 17 (E17) *SD* rat brains and cultured in Neurobasal A (Invitrogen, 21103-049). For RNAi experiments, isolated cortical neurons were first plated for about 3 days and then transfected with siRNA-Mate (GenePharma). The siRNAs for *TRPC6* (sense-1: CGGUGGUCAUCAACUACAATT; antisense-1: UUGUAGUUGAUGACCACCGTT; sense-2: GCUUGACUUUGGAAUGUUATT; antisense-2: UAACAUUCCAAAGUCAAGCTT), *TRPC5* (sense: AACGCCUUCUCCACGCUCUUU; antisense: AAAGAGCGUGGAGAAGGCGUU), *Ca*_*v*_*1.2* (sense: GCCGAAAUUACUUCAAUAUTT; antisense: AUAUUGAAGUAAUUUCGGCTT), *Ca*_*v*_*3.1* (sense: GGAGGAGAUUGAGGUCAAUTT, antisense: AUUGACCUCAAUCUCCUCCTT), *Ca*_*v*_*3.3* (sense: GCCCUUAAGUACUGCAACUTT; antisense: AGUUGCAGUACUUAAGGGCTT) and negative control (sense: GCACACCGAAGGAUAUUCUTT; antisense: AGAAUAUCCUUCGGUGUGCTT) were synthesized by GenePharma.

### Ca^2+^ imaging

Determination of cytosolic Ca^2+^ elevation was conducted mainly according to previously reported^13^. Briefly, transiently transfected HEK293 or HEK293TRPC6 cells on 12 mm coverslips were washed three times with MEM, and loaded with 2 μM Fura 2-AM (Molecular Probes) for 25 min at 37 °C in the dark. The cells were then washed with Hepes-buffered saline solution (HBSS: 120 mM NaCl, 5.3 mM KCl, 0.8 mM MgSO_4_, 1.8 mM CaCl_2_, 10 mM glucose, 20 mM Hepes, pH7.4) and imaged with a Nikon eclipse TE2000-e microscope with excitations at 340 and 380 nm and detection of emissions at 500 nm for Fura 2-AM. The F340/F380 ratio, which increases as a function of intracellular Ca^2+^ concentration, was captured at 6 s intervals.

### C99 expression and purification

The coding sequence^6^ of C99 was cloned into PET-28a(+) vector. To determine the optimal condition for the expression of the recombinant protein, the His-tagged C99 fusion protein expression was induced with 1 mM IPTG for 0, 2, 4, 6 and 8 h at 37 °C in BL21 *Escherichia coli* (Novagen). Then, the bacteria induced for 8 h were collected by centrifugation and the soluble proteins were extracted by BugBuster Master Mix (Novagen) according to the manufacturer's instructions. The recombinant protein was purified by Ni-column (QIAGEN) and reconstituted in TBS (25 mM Tris-HCl, 150 mM NaCl, pH 7.2) at a concentration of 0.5 μg μl^−1^.

### *In vitro* purified C99 cleavage assay

The purified C99 recombinant protein was used as the substrate of γ-secretase. HEK293 cells were homogenized in lysis buffer (10 mM Tris-HCl, 1 mM EGTA, 1 mM EDTA, pH7.4) and centrifuged at 1,000*g* for 15 min. The supernatant was further centrifuged at 13,200 r.p.m. at 4 °C for 15 min. γ-Secretase was reconstituted by resolving the pellets in lysis buffer with 0.2% (w/v) CHAPSO. The *in vitro* C99 cleavage assay was performed by incubating 20 μg of γ-secretase fraction and 2 μg C99-His recombinant protein in 100 μl reaction buffer (50 mM Tris-HCl, 2 mM EDTA, 0.25% CHAPSO, pH 6.8) at 37 °C for 4 h. Then, Aβ levels were determined by ELISA.

### Antibodies and drugs

The following antibodies were used: TRPC6 from Alomone (ACC-017)^12^, Sigma (PRS3889)^19^ and SAB (21403), TRPC5 from Sigma (ACC-020)^48^, Ca_v_1.2 from Millipore (AB5156), Ca_v_3.1 (ACC-021) and Ca_v_3.3 (ACC-009) from Alomone, APP from Invitrogen (13-0200, clone LN27) and Sigma (A8717), 6E10 from Chemicon (MAB1560) and Signet (SIG-39300), MOAB-2 from Kerafast (EB2001), ADAM10 from Abcam (Ab1997), clusterin from Santa Cruz (sc-6420), BACE1 from Millipore (MAB5308), insulin-degrading enzyme from EMD Biosciences (PC730), neprilysin from Epitomics (2569–1), PS1 from Chemicon (MAB5232), nicastrin from Sigma (MAB5232), presenilin enhancer 2 from Invitrogen (36–7100), E-Cadherin from Abcam (ab53033), N-Cadherin from BD (610921), cleaved-Notch from Cell Signalling Technology (2421 L), Notch from Proteintech (10062-2-AP), EEA1 from BD (610457), Calnexin from Enzo (ADI-SPA-860-F), GM130 from BD (610823), Bip from BD (610979), Cathepsin D from Santa Cruz (sc-6487), Myc from Chemicon (05–724), HA from Sigma (H6908) and Genscript (A00168), and His from Abmart (M30111). OAG (O6754), SKF96365 (S7809) and L685,458 (L1790) were from Sigma.

### Peptide injection

TAT fusion peptides were synthesized by ChinaPeptides. Male littermate *APP/PS1* mice were injected intra-peritoneally with 200 μl 2.5 mM TAT or TAT-TM2 and sacrificed for Aβ quantification 3 h after injection in a blinded manner.

### Fractionation

Fractionation was carried out as reported previously^49^. Briefly, mouse brain was homogenized in 1 ml homogenization buffer (HB: 20 mM Tris-HCl, pH 7.4, 250 mM sucrose, 1 mM EGTA, 1 mM EDTA and protease inhibitors) by 20 strokes followed by 10 passages through a 21-gauge needle. Lysates were centrifuged at 3,000 r.p.m. for 15 min at 4 °C to generate the post-nuclear supernatant. The post-nuclear supernatant was mixed 1:1 with OptiPrep (Sigma, D1556, 60% iodixanol in water). The resultant 2 ml 30% iodixonol mixture was placed at the bottom of an ultracentrifuge tube and was overlaid successively with 1 ml 25, 20, 17.5, 15, 12.5, 10, 7.5, 5 and 2.5% iodixonol in cold HB. The gradients were centrifuged for 20 h at 27,000 r.p.m. at 4 °C using a Beckman SW41 rotor. The resulting gradient was collected in 500 μl fractions from the top of the ultracentrifuge tube and prepared for western blot analysis. For each blot, two gels with 11 lanes were run in parallel and the results were merged together.

### Plasmids construction

Plasmids encoding the following fragments of mouse TRPC6 fused with HA tag C-terminally were cloned into AscI/ClaI sites of pCAGGS vector: 1–405, 725–930, 389–510, 389–930, 437–633 and 508–633 aa. The PCR primers were as follows: 1–405S, ATAGGCGCGCCACCATGAGCCAGAGCCCGAG; 1–405AS, GTAATCGATTCAAGCGTAATCTGGTACGTCGTATGGGTACTTCACTGCCATGG; 725–930S, ATAGGCGCGCCACCATGATCAATAGTTCATTC; 725–930AS, GTAATCGATTCAAGCGTAATCTGGTACGTCGTATGGGTATCTGCGGCTTTCCTC; 389–510S, ATAGGCGCGCCACCATGATATGGTATGAGAACC; 389–510AS, GTAATCGATTCAAGCGTAATCTGGTACGTCGTATGGGTATTTACATTCAGCCCAT; 389–930S, ATAGGCGCGCCACCATGATATGGTATGAGAACC; 389–930AS, GTAATCGATTCAAGCGTAATCTGGTACGTCGTATGGGTATCTGCGGCTTTCCTCC; 437–633S, ATAGGCGCGCCACCATGGGACCGTTCATGAAG; 437–633AS, GTAATCGATTCAAGCGTAATCTGGTACGTCGTATGGGTAGATATCTTTCACTGTTC; 508–633S, ATAGGCGCGCCACCATGGAATGTAAAGAAATC; 508–633AS, GTAATCGATTCAAGCGTAATCTGGTACGTCGTATGGGTAGATATCTTTCACTGTTC.

The TM2 domain of mouse TRPC6 was mutated by the following primer pairs and then cloned into AscI/ClaI sites of pCAGGS vector. *C6-mut1*: GAGATGCTCATTATATCCGACGACATAGGCATGGACGACGCTGAATGTAAAGAAATC, GATTTCTTTACATTCAGCGTCGTCCATGCCTATGTCGTCGGATATAATGAGCATCTC; C6-mut2:GAGATGCTCATTATATCCGCTGCTATAGGCATGGCTGCTGCTGAATGTAAAGAAATCTG, CAGATTTCTTTACATTCAGCAGCAGCCATGCCTATAGCAGCGGATATAATGAGCATCTC; *C6-mut3*: CCGCCGCCCACGCCGTGTTCAAGATGTTCCCCGAATGTAAAGAAATCTGGACTC, AGAAGGTGATGAACAGGCCCAGCAGCACCATTTTCATCCTGAACAGCTGCCTTGC;*C6-mut4*: CCTGATGGAGATGTGGTCCTTCTGCTCCACCGAATGTAAAGAAATCTGGACTC, ATGATGGACCACACGATGCCCATGATCCAGGCTTTCATCCTGAACAGCTGCCTTG.

FAD-linked APP variants were mutated from WT APP with following primer pairs and then cloned into AscI/ClaI sites of pCAGGS vector.

*APP-I716F*: GTTGTCATAGCGACAGTGTTCGTCATCACCTTGGTGATGC, GCATCACCAAGGTGATGACGAACACTGTCGCTATGACAAC; *APP-V717F*: GTCATAGCGACAGTGATCTTCATCACCTTGGTGATGCTG, CAGCATCACCAAGGTGATGAAGATCACTGTCGCTATGAC.

### Statistical analysis

All data excluding behavioural experiment were analysed by two-tailed Student's *t*-test for two groups, and one way ANOVA with Newman–Keuls *post hoc* test for more than two groups using GraphPad Prism. For all experiments, the sample sizes were chosen according to previous literatures in the field. For all, *P<0.05, **P<0.01, ***P<0.001. All data were presented as mean±s.e.m.

## Additional information

**How to cite this article:** Wang, J. *et al.* TRPC6 specifically interacts with APP to inhibit its cleavage by γ-secretase and reduce Aβ production. *Nat. Commun.* 6:8876 doi: 10.1038/ncomms9876 (2015).

## Supplementary Material

Supplementary Information Supplementary Figures 1-18
