# Supplementary material for: TRPC6 specifically interacts with APP to inhibit its cleavage by γ-secretase and reduce Aβ production
Source: Nat Commun. 2015 Nov 19;6:8876. doi: 10.1038/ncomms9876 (PMC4696454; doi:10.1038/ncomms9876)
Supplement: Supplementary Information — Supplementary Figures 1-18 [file ncomms9876-s1.pdf]

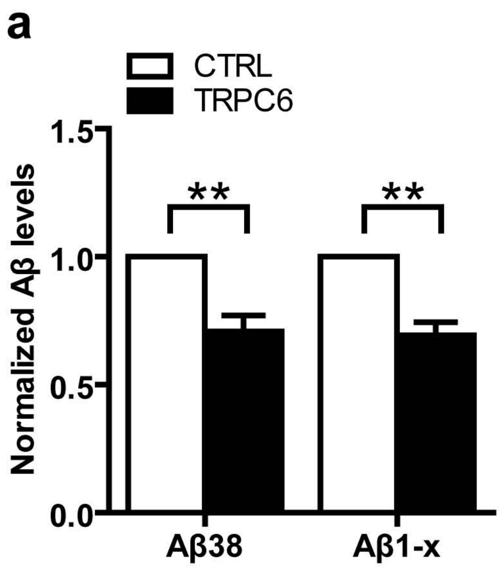

Supplementary Figure 1. TRPC6 regulated Aβ38 and Aβ1-x levels in cultured cells.

Supplementary Figure 1 TRPC6 regulated Aβ38 and Aβ1-x levels in cultured cells. (**a**) ELISA examination of Aβ38 and Aβ1-x levels in the medium of HEK293APP stable cells transfected with *TRPC6* for 2 days (n=5). CTRL, transfection with *YFP*. Data were presented as means ± s.e.m. of indicated numbers of independent experiments. Two-tailed Student's t test was performed. **P<0.01 vs. CTRL.

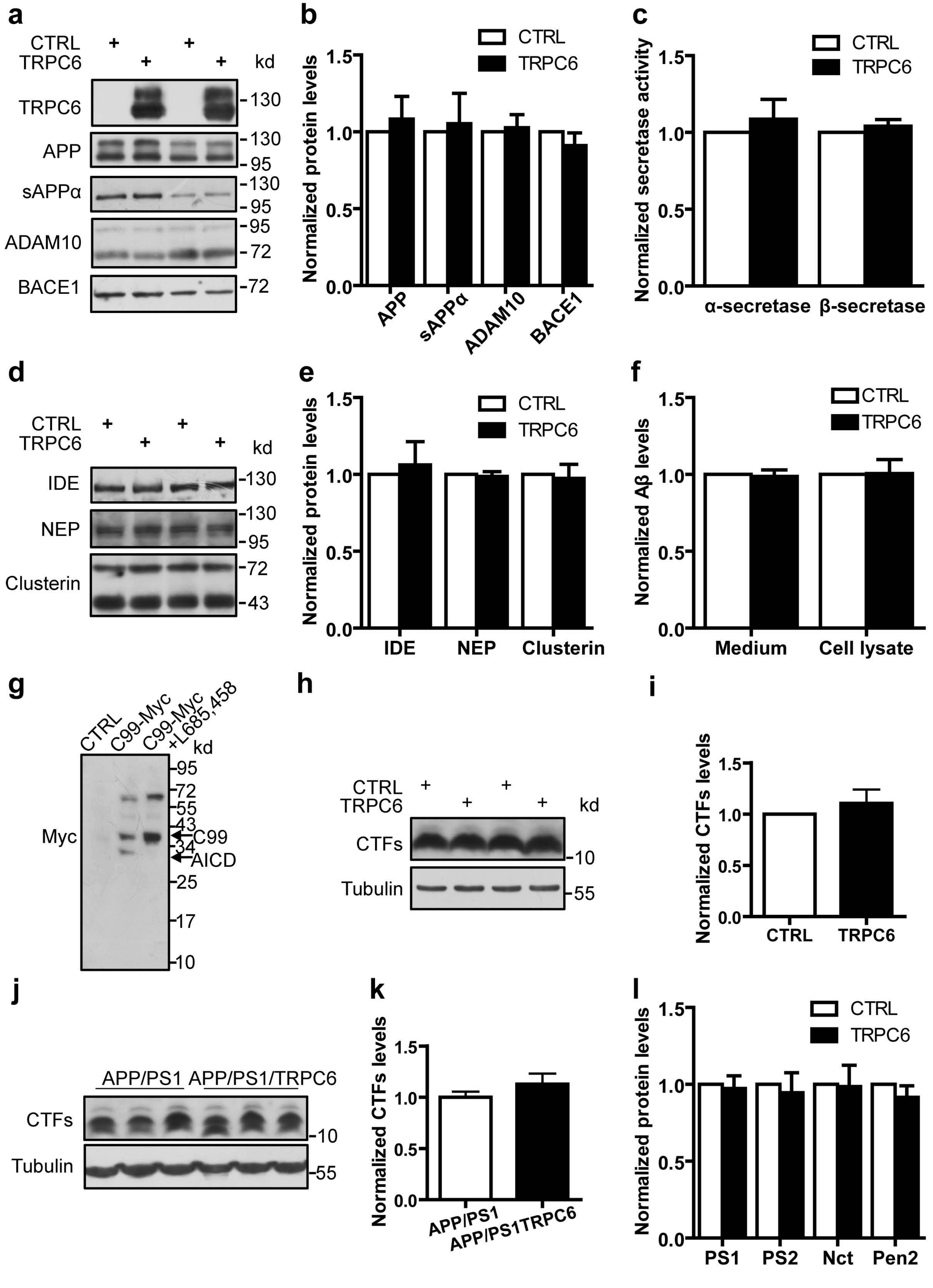

Supplementary Figure 2. TRPC6 did not affect α-/β- secretase cleavage of APP or Aβ clearance.

Supplementary Figure 2 TRPC6 did not affect α-/β- secretase cleavage of APP or Aβ clearance. (**a**) Immunoblots of APP, sAPPα, ADAM10 and BACE1 in HEK293APP cells transfected with *TRPC6* for 2 days. (**b**) Quantification of indicated protein levels (n=4-7). (**c**) α- or β- secretase activity in the lysates of HEK293APP cells transfected with *YFP* or *TRPC6* for 2 days in the in vitro fluorogenic substrate assay (n=5 in duplication). (**d**) Immunoblot analysis of the expression levels of insulin-degrading enzyme (IDE), neprilysin (NEP) or clusterin in HEK293APP cells transfected with *TRPC6* for 2 days. (**e**) Quantification of indicated protein levels (n=4-5). (**f**) ELISA examination of Aβ levels in the medium and cell lysates 12 hours after addition of 1 μg ml^{-1} Aβ to the culture medium of HEK293 cells (n=3). (**g**) Immunoblot analysis of HEK293 cells transfected with *C99-Myc* for 1 day and treated with 10 μM L685,458 for 12 hours. Arrows indicated C99 and AICD. Immunoblot analysis (**h**) and quantification (**i**) of CTFs levels in the HEK293APP cells transfected with *YFP* or *TRPC6* for 2 days (n=3). Immunoblot analysis (**j**) and quantification (**k**) of CTFs levels in the brain lysates of 11 month male *APP/PS1* or *APP/PS1/TRPC6* mice (n=5). (**l**) Quantification of indicated protein levels (n=5). CTRL, transfection with *YFP* or pcDNA3.1. Data were presented as means ± s.e.m. of indicated numbers of independent experiments. Two-tailed Student's t test was performed.

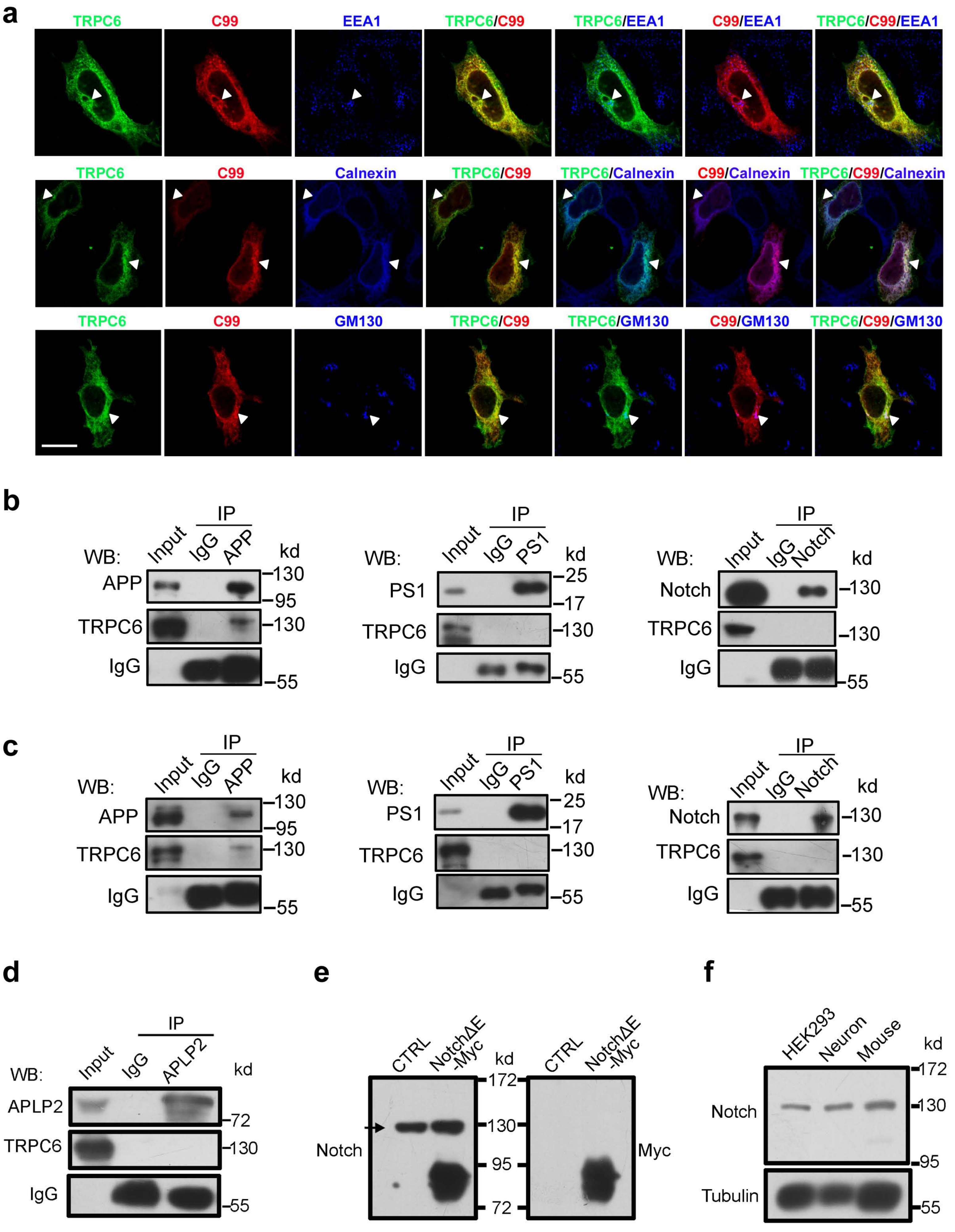

Supplementary Figure 3. TRPC6 specifically interacted with APP (C99), but not with PS1 or Notch.

Supplementary Figure 3 TRPC6 specifically interacted with APP (C99), but not with PS1 or Notch. (**a**) Immunocytochemical analysis of HEK293 cells transfected with *C99-Myc* and *TRPC6-HA* for 3 days. Arrow heads indicated the colocalized signals. Scale bar, 20 μm. Immunoprecipitation of cell lysates from HEK293 cells (**b**) or primary cultured cortical neurons (**c**) with antibody against APP, PS1 or Notch, and immunoblot analysis using the indicated antibodies. (**d**) Mouse brain lysates precipitated with the antibody against APLP2, and immunoblotted with indicated antibodies. (**e**) Immunoblot analysis of HEK293 cells transfected with *Notch△E-Myc* for 2 days with indicated antibodies. Arrow indicated endogenous Notch. (**f**) Immunoblot analysis of lysates of HEK293 cells, primary cultured neurons and mouse brain with indicated antibodies. CTRL, transfection with *YFP*.

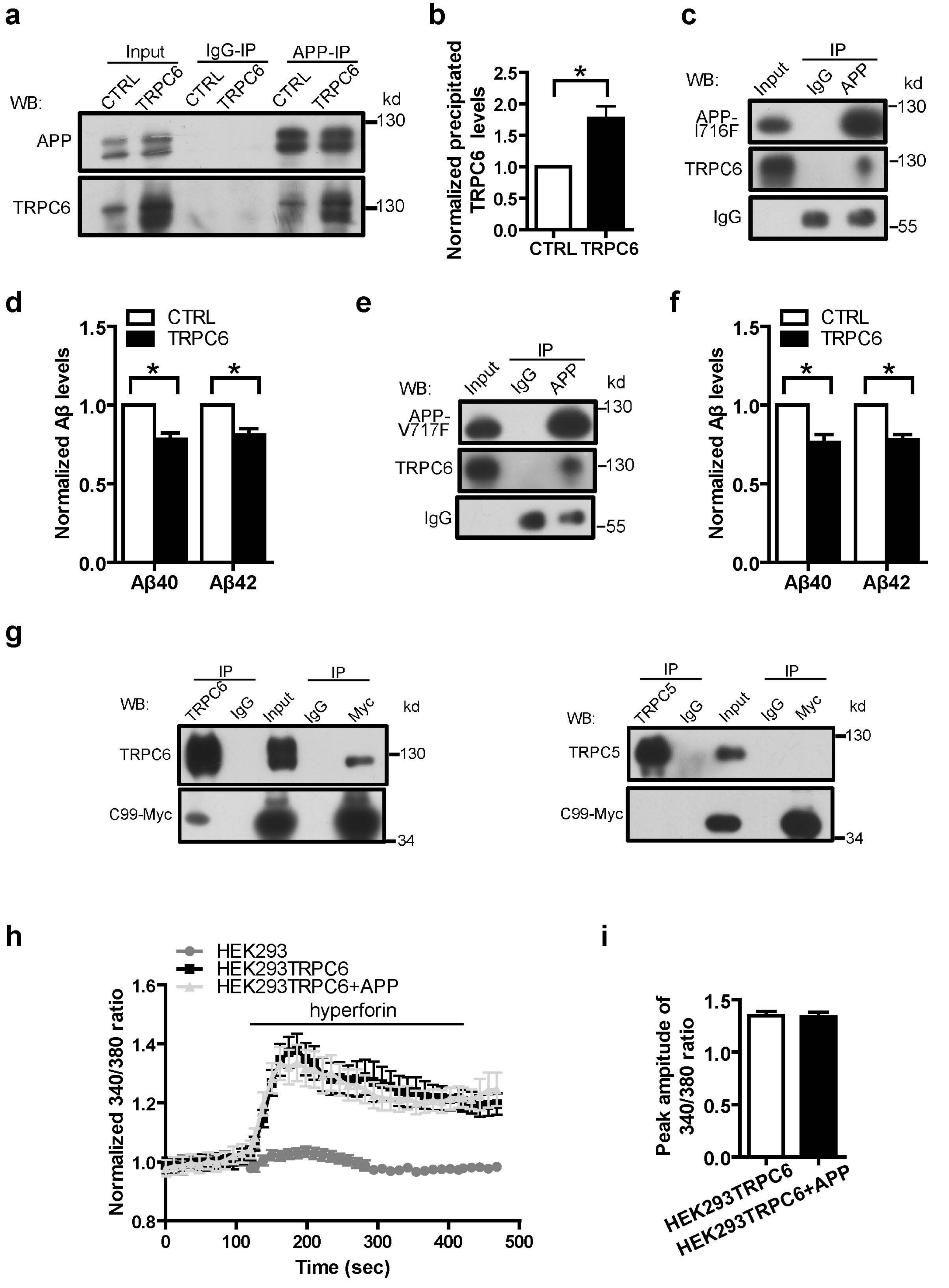

Supplementary Figure 4. TRPC6 interacted with APP (C99).

Supplementary Figure 4 TRPC6 interacted with APP (C99). (**a**) Lysates of HEK293APP stable cells transfected with *YFP* or *TRPC6* for 2 days precipitated with APP antibody, and immunoblotted with indicated antibodies. (**b**) Quantification of precipitated TRPC6 levels (n=4). (**c**) Lysates of HEK293TRPC6 stable cells transfected with *APP-I716F* for 2 days precipitated with APP antibody, and immunoblotted with indicated antibodies. (**d**) Aβ levels in the medium of HEK293 cells transfected with *APP-I716F* for 1 day and further transfected with *TRPC6* for 2 days (n=3-4). (**e**) Lysates of HEK293TRPC6 stable cells transfected with *APP-V717F* for 2 days precipitated with APP antibody, and immunoblotted with indicated antibodies. (**f**) Aβ levels in the medium of HEK293 cells transfected with *APP-V717F* for 1 day and further transfected with *TRPC6* for 2 days (n=3). (**g**) Lysates of HEK293C99 stable cells transfected with *TRPC5* or *6* for 2 days precipitated with Myc, TRPC5 or 6 antibody, and immunoblotted with indicated antibodies. (**h**) Representative traces and (**i**) quantification of Ca^{2+} entry induced by 5 μM hyperforin in HEK293TRPC6 stable cells transfected with CTRL or *APP* (n=45-75 cells from 3 independent experiments). CTRL, transfection with *YFP* or pcDNA3.1. Data were presented as means ± s.e.m. of indicated numbers of independent experiments. Two-tailed Student's t test was performed. *P<0.05 vs. CTRL.

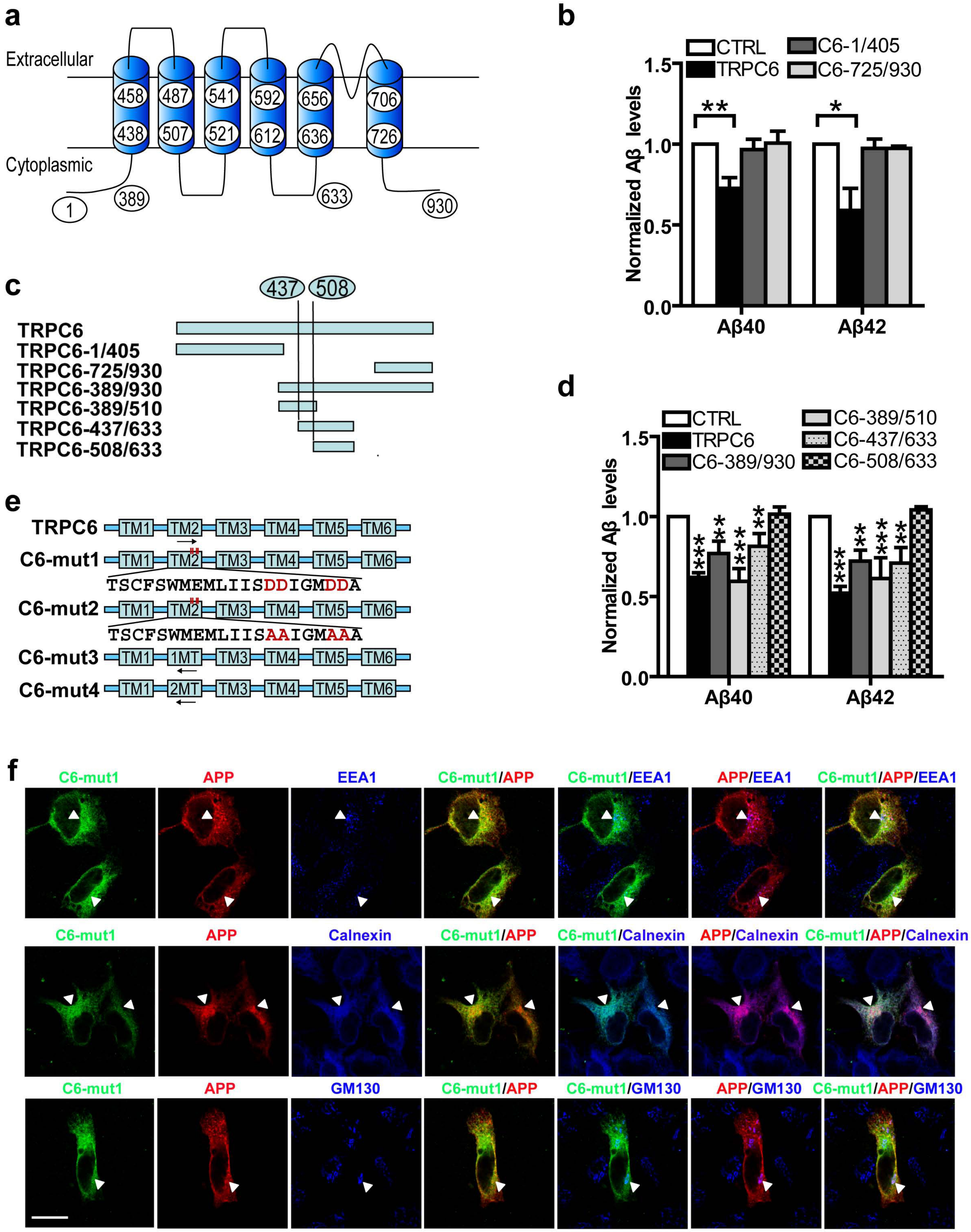

Supplementary Figure 5. Illustrations and effects of TRPC6 mutational constructs.

Supplementary Figure 5 Illustrations and effects of TRPC6 mutational constructs. (**a**) Schematics of the topographic structure of TRPC6. (**b**) ELISA examination of Aβ levels in the medium of HEK293APP stable cells transfected with *TRPC6*, *TRPC6-1/405* or *TRPC6-725/930* for 2 days (n=4-8). (**c**) Schematics of TRPC6 mutational constructs. (**d**) The Aβ levels in the medium of HEK293APP cells transfected with indicated fragments of *TRPC6* for 2 days (n=3-5). (**e**) Schematics of TRPC6 mutations within second transmembrane domain. The TM2 domain was mutated by point mutations, replacement with TM1 domain or reversal of TM2 sequence. (**f**) Immunocytochemical analysis of HEK293 cells transfected with *APP-Myc* and *C6-mut1-HA* for 3 days. Arrow heads indicated the colocalized signals. Scale bar, 20 μm. CTRL, transfection with *YFP*. Data were presented as means ± s.e.m. of indicated numbers of independent experiments. One way ANOVA with Newman-Keuls post hoc test was performed. $^*P<0.05$, $^{**}P<0.01$, $^{***}P<0.001$ vs. CTRL.

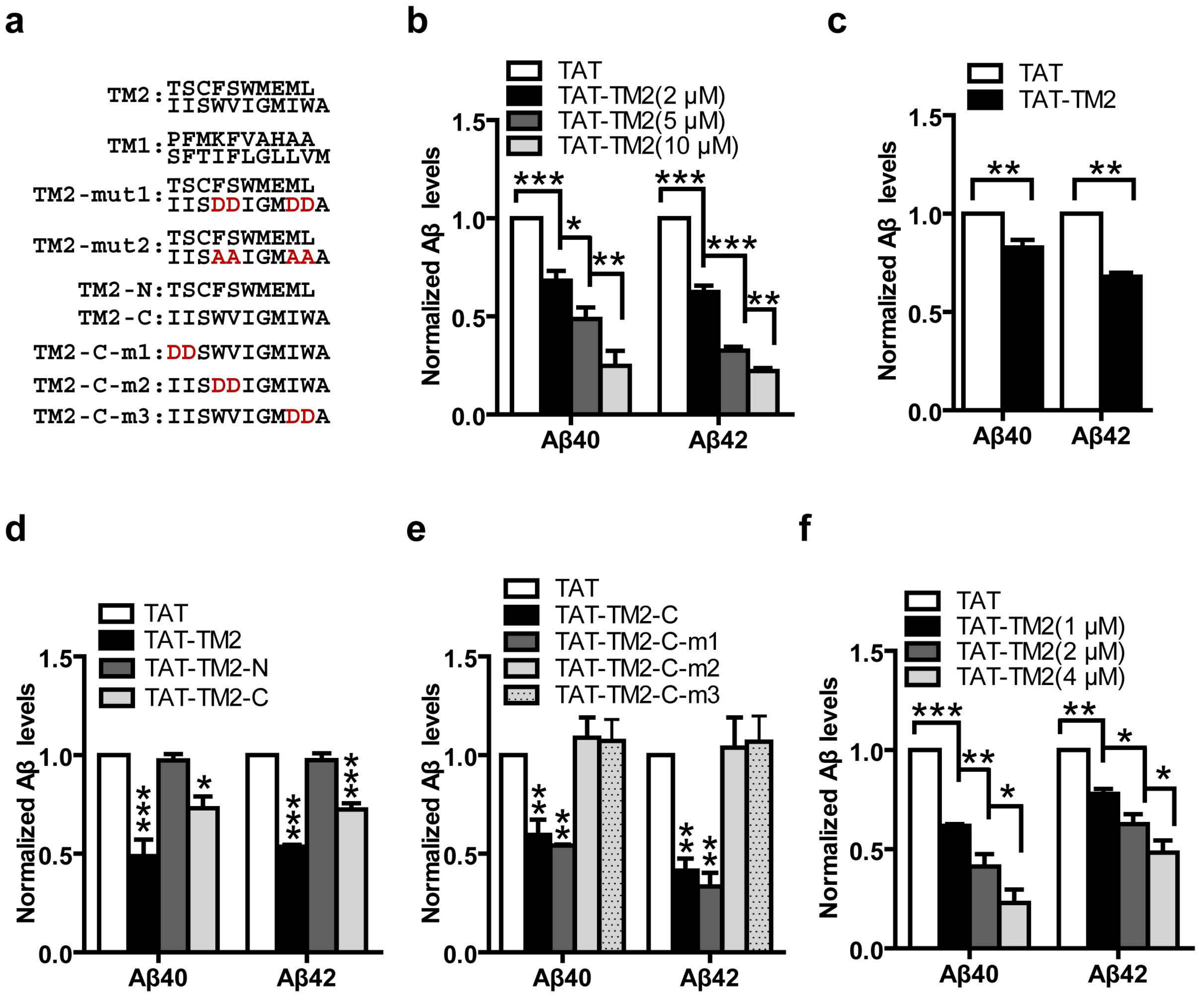

Supplementary Figure 6. TAT-TM2 reduced Aβ levels in cultured cell and in the in vitro cleavage assay.

Supplementary Figure 6 TAT-TM2 reduced Aβ levels in cultured cells and in the in vitro cleavage assay. (**a**) Amino acid sequences of different peptides. (**b**) ELISA examination of Aβ levels in the medium of HEK293APP cells treated with indicated concentrations of TAT-TM2 for 12 hours (n=4-6)**.** (**c**) Aβ levels in the medium of primary cultured cortical neurons treated with 0.2 μM TAT-TM2 for 24 hours (n=3-6). (**d**) Aβ levels in the medium of HEK293APP cells treated with 5 μM of indicated peptides for 12 hours **(**n=3-5**).** (**e**) Aβ levels in the medium of HEK293APP cells treated with 5 μM of indicated peptides for 12 hours (n=3). (f) The Aβ levels in the in vitro C99 cleavage assay in the presence of indicated concentrations of TAT-TM2 for 2 hours (n=4-5). Data were presented as means ± s.e.m. of indicated numbers of independent experiments. Two-tailed Student's t test was performed for two groups, and one way ANOVA with Newman-Keuls post hoc test was performed for more than two groups. *P<0.05, **P<0.01, ***P<0.001 vs. TAT.

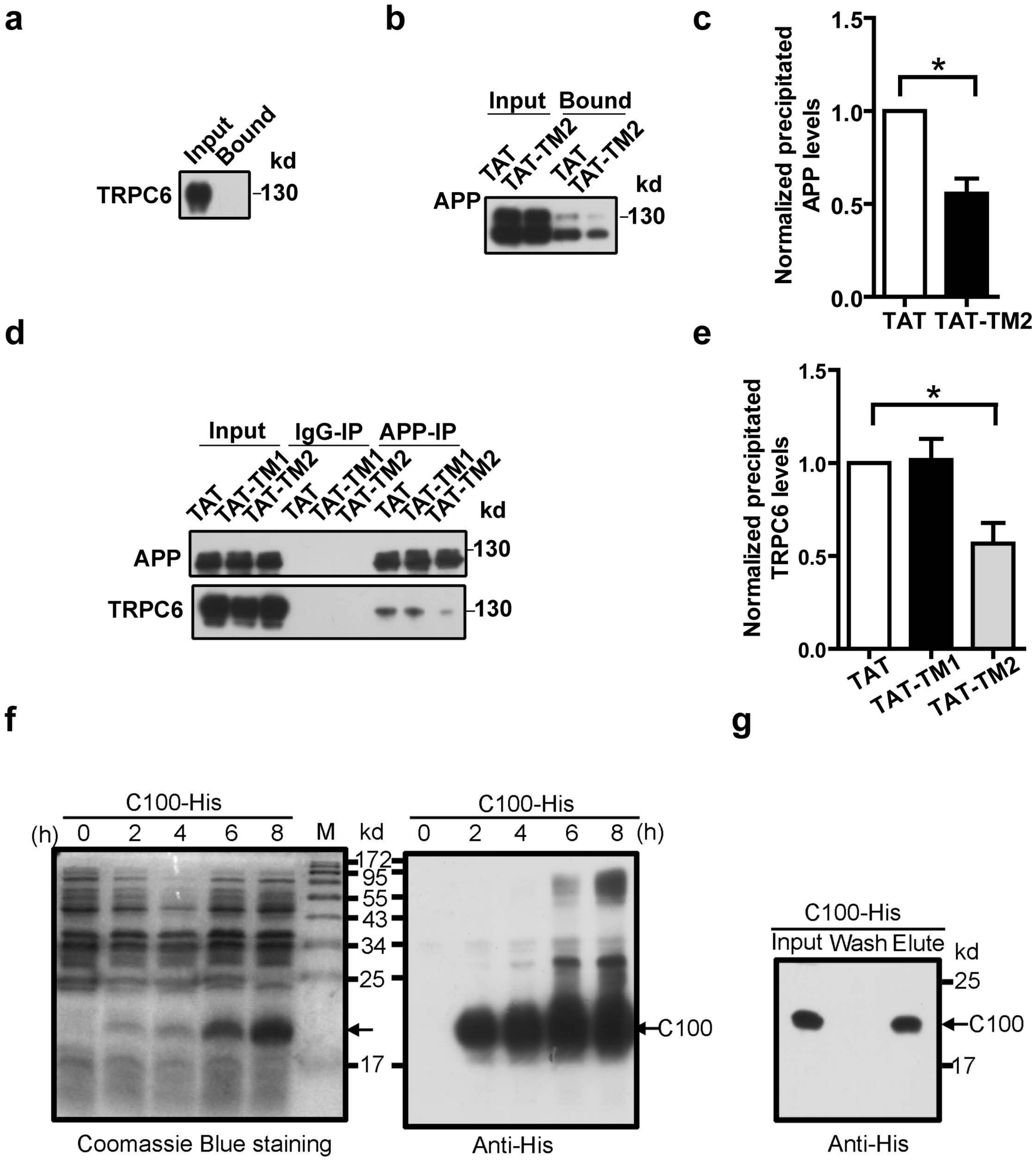

Supplementary Figure 7. TAT-TM2 bound to APP.

Supplementary Figure 7 TAT-TM2 bound to APP. (**a**) Lysates of HEK293TRPC6 stable cells incubated with 5 μM TAT-TM2-bitoin for 6 hours precipitated with avidin beads and immunoblotted with indicated antibodies. (**b**) Lysates of HEK293APP stable cells incubated with 5 μM TAT-TM2-bitoin in the presence of 5 μM TAT or TAT-TM2 for 6 hours precipitated with avidin beads and immunoblotted with indicated antibodies. (**c**) Quantification of the precipitated APP levels shown in **b** (n=3). (**d**) Lysates of HEK293TRPC6 stable cells transfected with *APP* for 2 days, treated with 5 μM indicated peptides for 6 hours and then precipitated with APP antibody, and immunoblotted with indicated antibodies. (**e**) Quantification of the precipitated TRPC6 levels shown in **d** (n=3). (**f**) Coomassie blue staining and immunoblot analysis of E.coli lysates after induction for indicated times. Arrow indicated C100. (**g**) Immunoblot analysis of C100-His after elution. Data were presented as means ± s.e.m. of indicated numbers of independent experiments. Two-tailed Student's t test was performed for two groups, and one way ANOVA with Newman-Keuls post hoc test was performed for more than two groups. *P<0.05 vs.TAT.

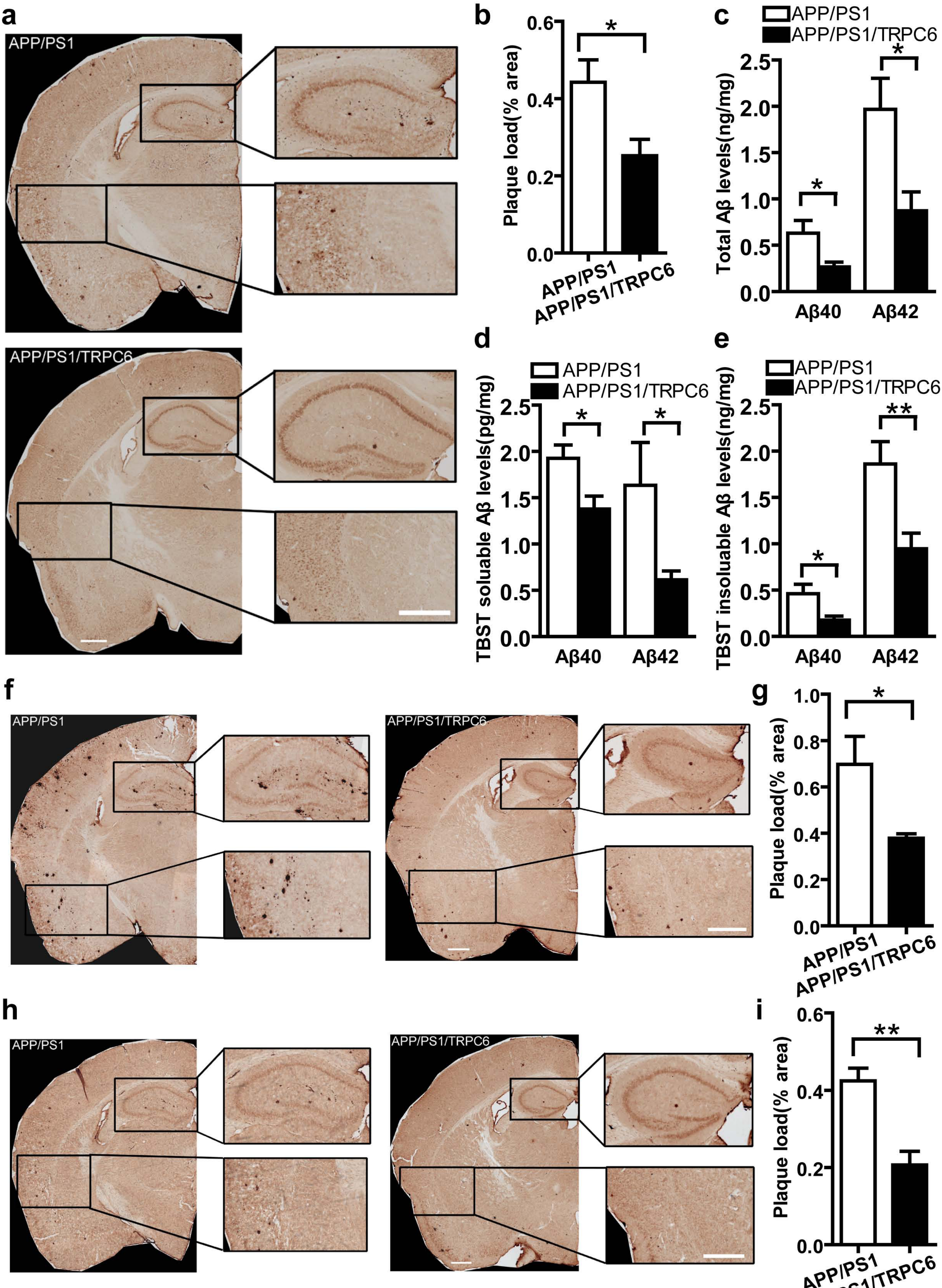

Supplementary Figure 8. The Aβ levels were decreased in APP/PS1/TRPC6 mice.

Supplementary Figure 8 The Aβ levels were decreased in *APP/PS1/TRPC6* mice. (**a**) Representative images of amyloid plaque immunostained with Aβ antibody (6E10) on brain sections from 6 month male *APP/PS1* and *APP/PS1/TRPC6* mice. Scale bar, 500 μm. (**b**) Quantification of the plaque load in indicated mouse brain sections shown in **a** (n=9). ELISA examination of total (**c**), TBST soluble (**d**) or insoluble (**e**) Aβ levels in the forebrain lysates of 6 month male indicated mice (n=10). (**f**) Representative images of amyloid plaque immunostained with Aβ-specific antibody MOAB-2 on brain sections from 6 month female *APP/PS1* and *APP/PS1/TRPC6* mice. Scale bar, 500 μm. (**g**) Quantification of the plaque load in indicated mouse brain sections shown in **f** (n=5). (**h**) Representative images of amyloid plaque immunostained with Aβ-specific antibody MOAB-2 on brain sections from 6 month male *APP/PS1* and *APP/PS1/TRPC6* mice. Scale bar, 500 μm. (**i**) Quantification of the plaque load in indicated mouse brain sections shown in **h** (n=4). Data were presented as means ± s.e.m.. Two-tailed Student's t test was performed. *P<0.05, **P<0.01 vs. *APP/PS1*.

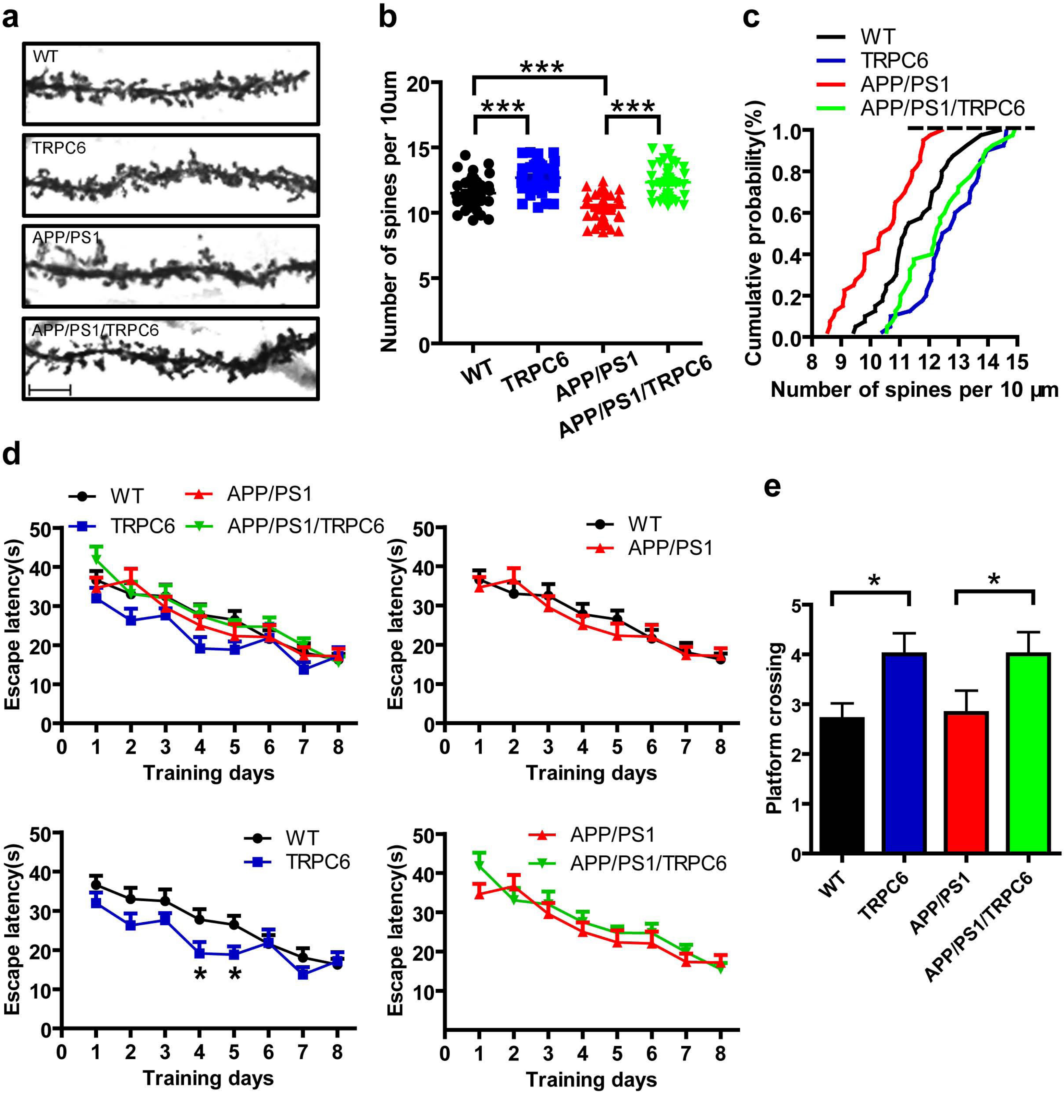

Supplementary Figure 9. APP/PS1/TRPC6 mice showed improved spatial memory ability at 6 month.

Supplementary Figure 9 *APP/PS1/TRPC6* mice showed improved spatial memory ability at 6 month. (**a**) Representative images of Golgi staining of hippocampal CA1 neurons in the four groups of mice at 6 month. Scale bar, 5 μm. (**b**) Quantification of spine number per 10 μm of dendrite (n=40 neurons from 4-6 mice). (**c**) Cumulative probability analysis of the spine density shown in **b**. (**d**) Escape latency of the four groups of mice at 6 month in the training session of Morris water maze (n=12-20). (**e**) Platform crossing number of the mice at 6 month in the probe session of Morris water maze (n=12-20). Data were presented as means ± s.e.m.. One way ANOVA with Newman-Keuls post hoc test was performed for spine quantification and repeated measures and multivariate analysis in general linear model with LSD post hoc test was performed for behavioral study. *$P<0.05$, ***$P<0.001$ vs. *WT* or *APP/PS1*.

Figure 1a

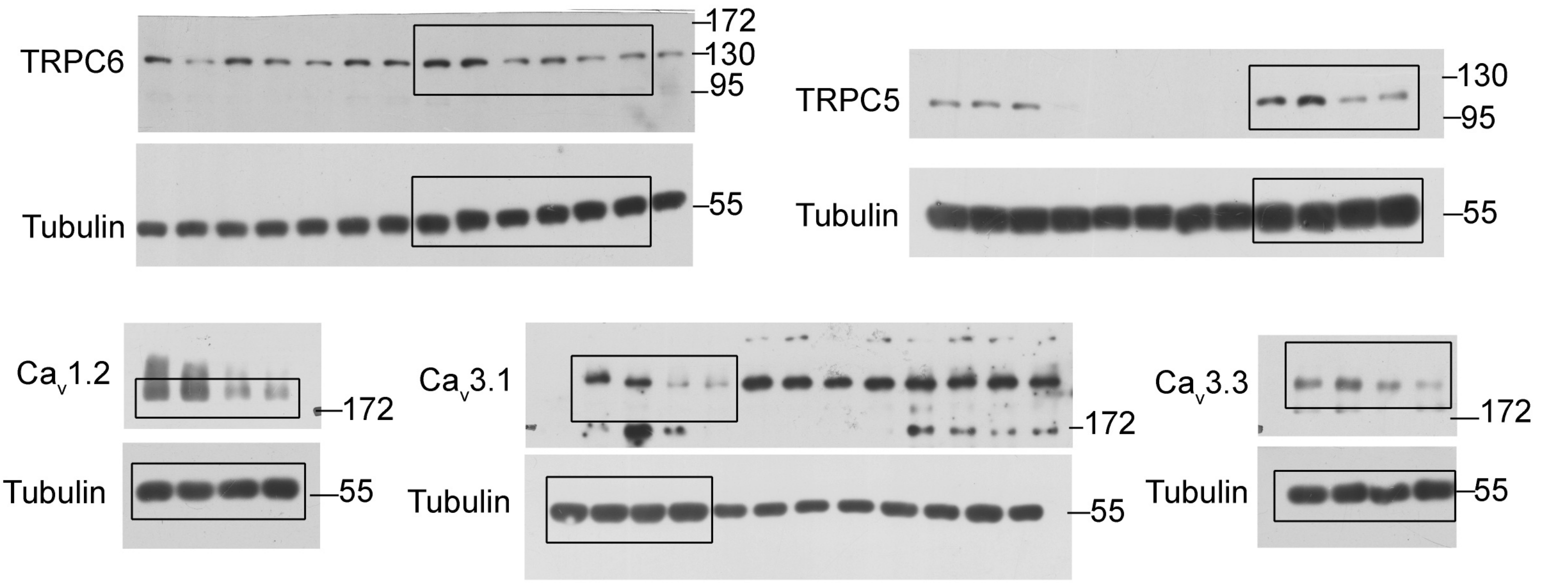

Figure 1c

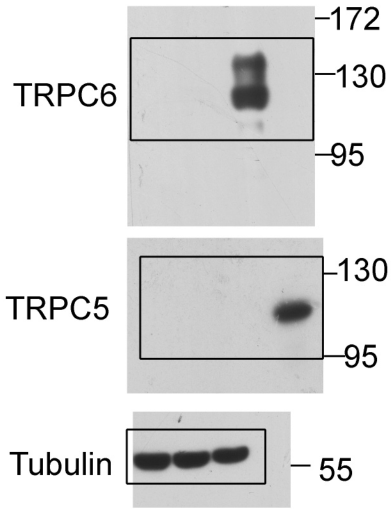

Figure 1e Figure 1d

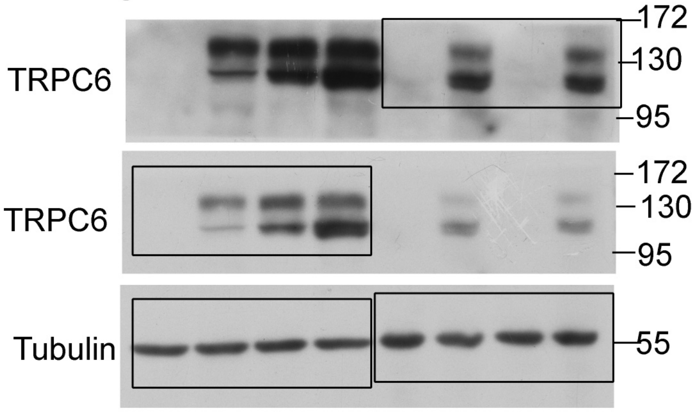

Supplementary Figure 10. Full scans of all blots in Figure 1.

Figure 2b

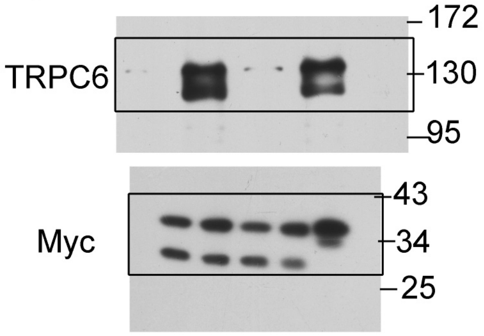

Figure 2c

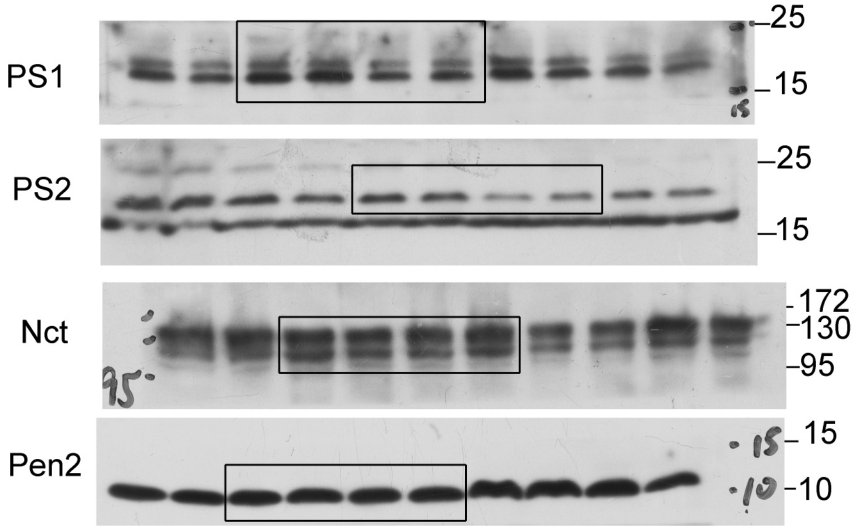

Figure 2f

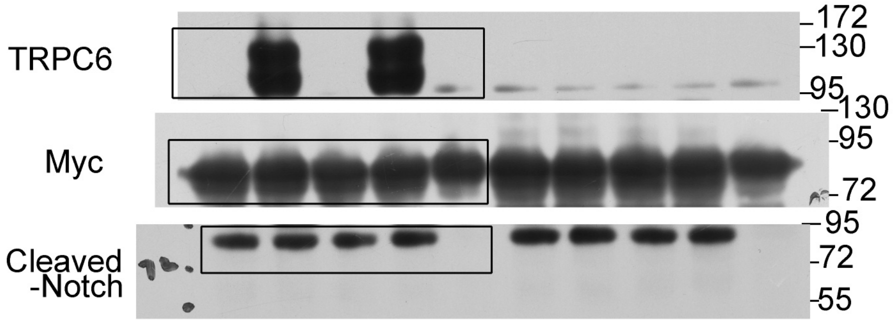

Figure 2h

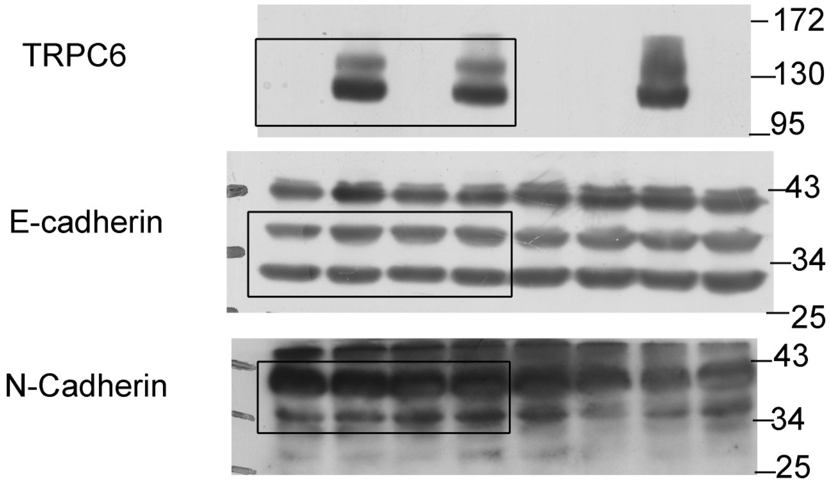

Supplementary Figure 11. Full scans of all blots in Figure 2.

Figure 3b

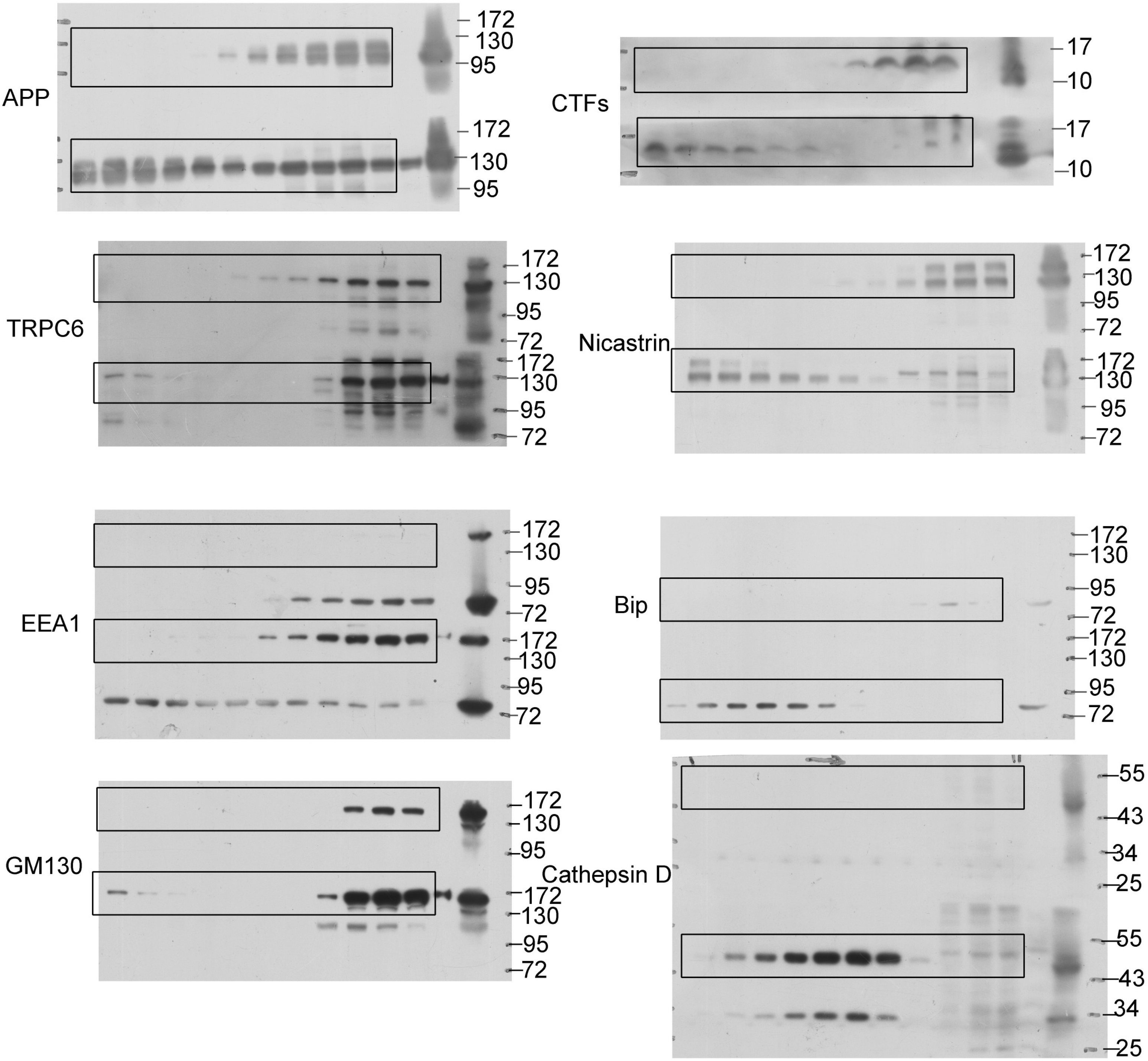

Supplementary Figure 12. Full scans of all blots in Figure 3b.

Figure 3c

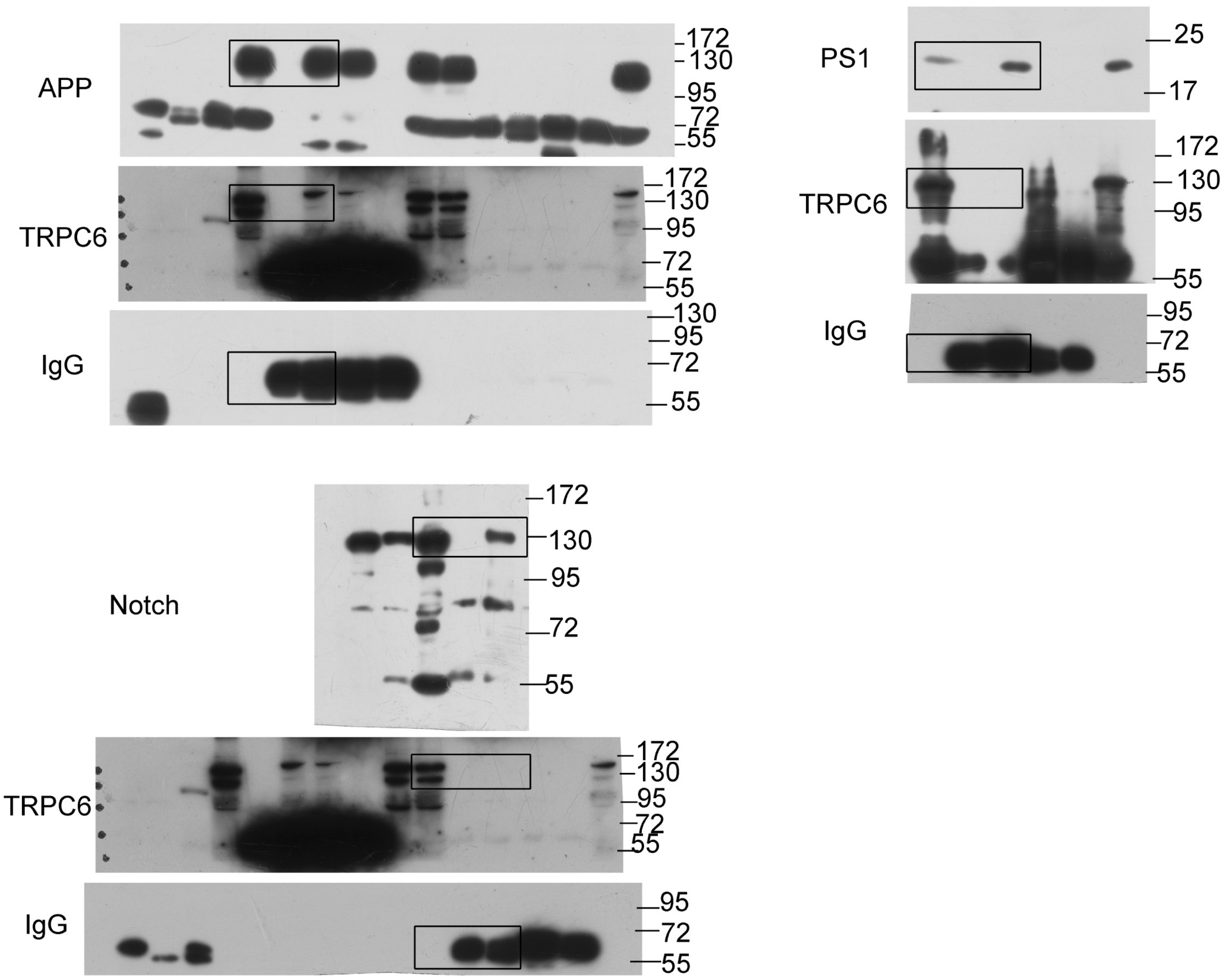

Figure 3d

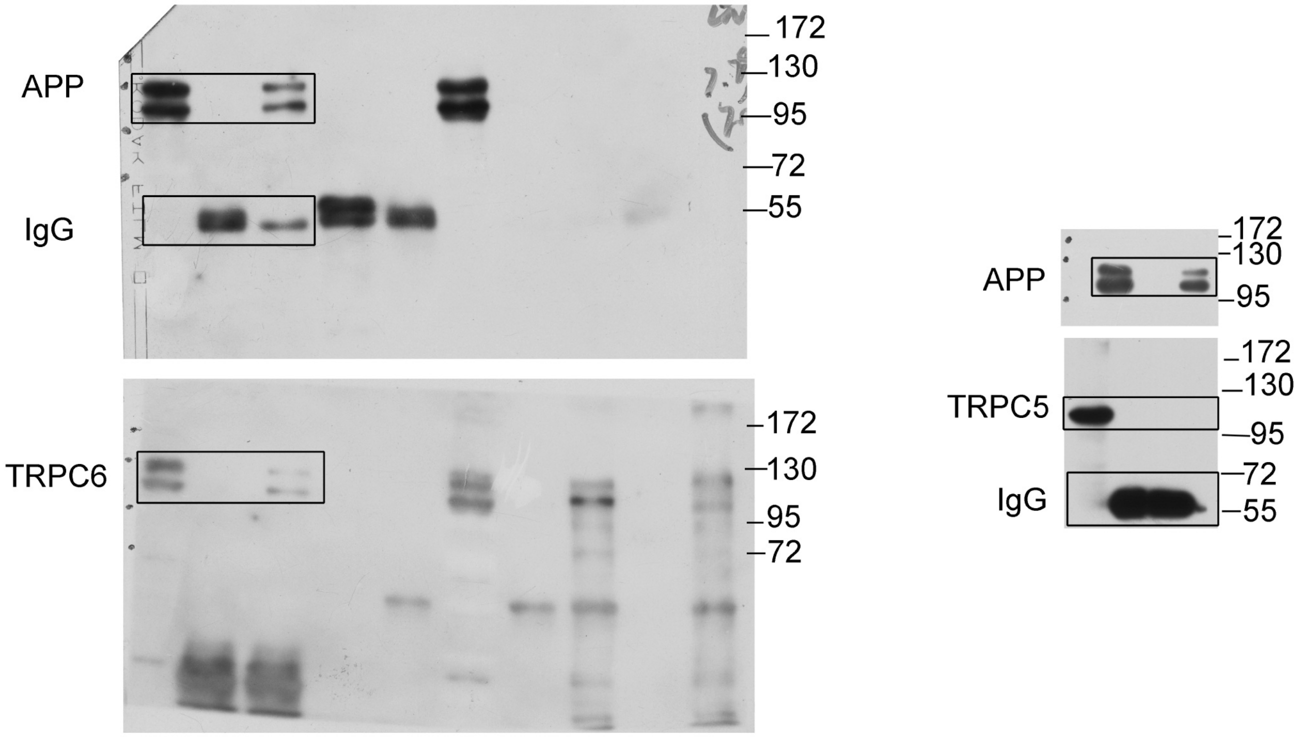

Supplementary Figure 13. Full scans of all blots in Figure 3c-d.

Figure 3e

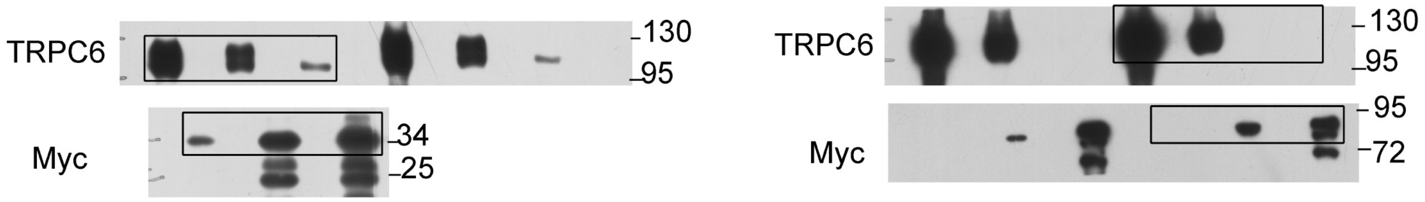

Figure 3f

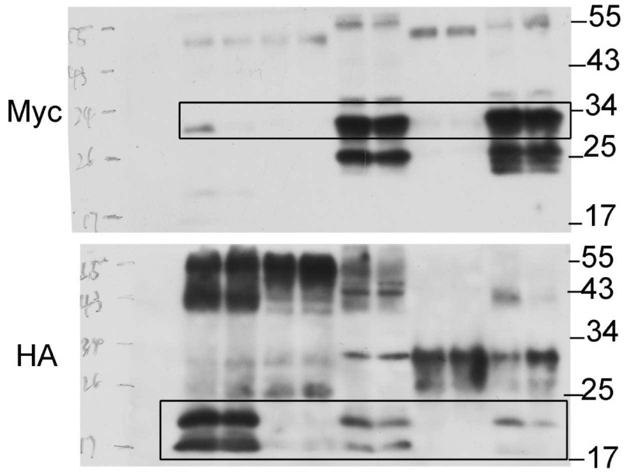

Figure 4b

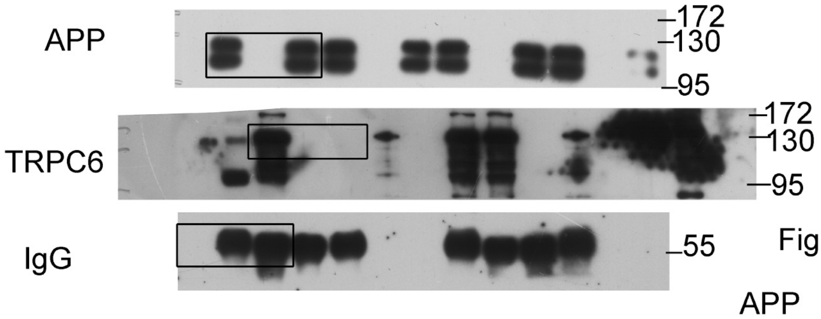

Figure 4d

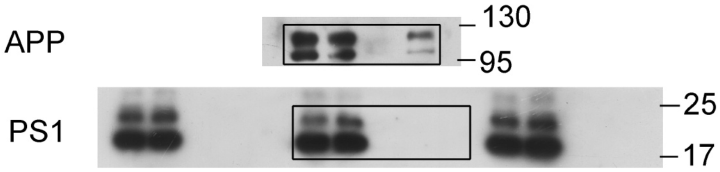

Figure 4h

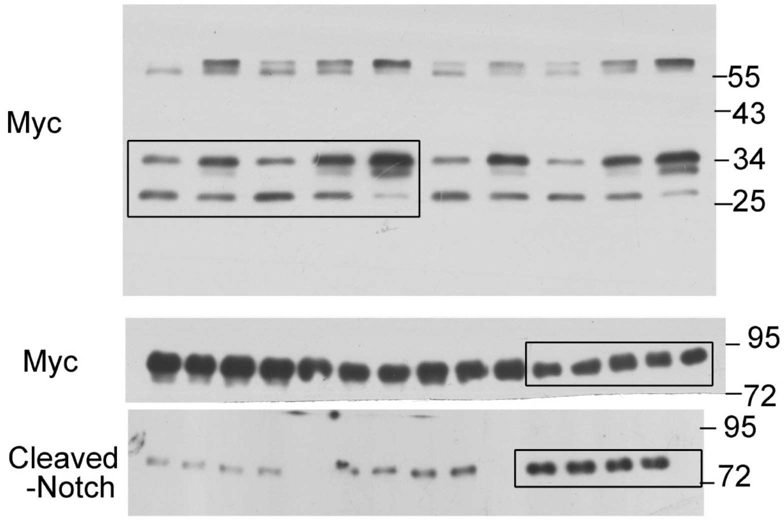

Supplementary Figure 14. Full scans of all blots in Figure 3e-f and 4.

Supplementary Figure 2a

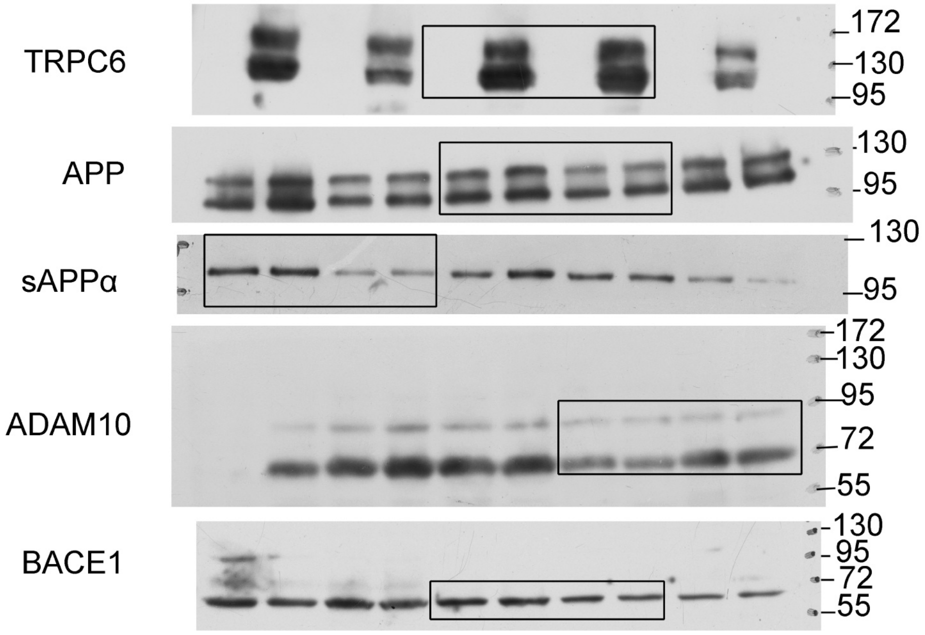

Supplementary Figure 2d

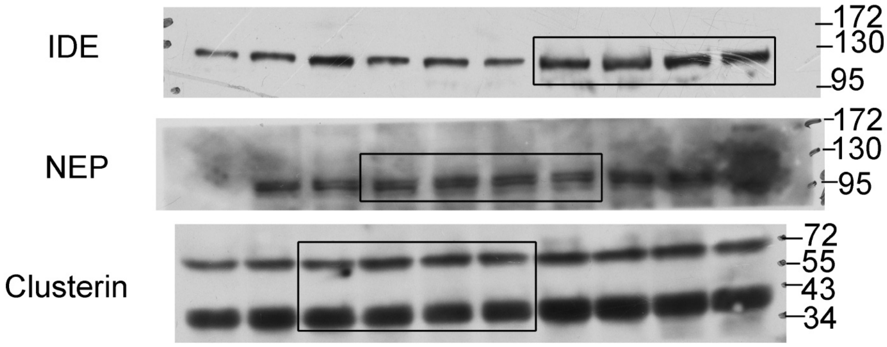

Supplementary Figure 2g

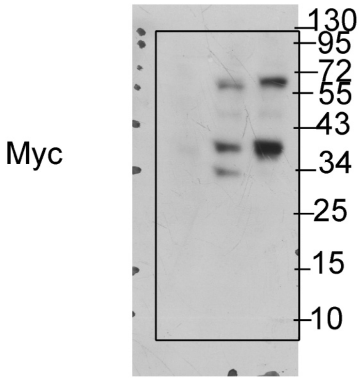

Supplementary Figure 2h

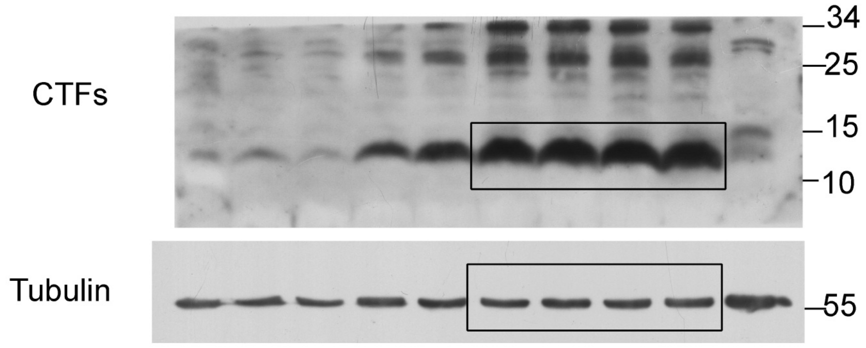

Supplementary Figure 2j

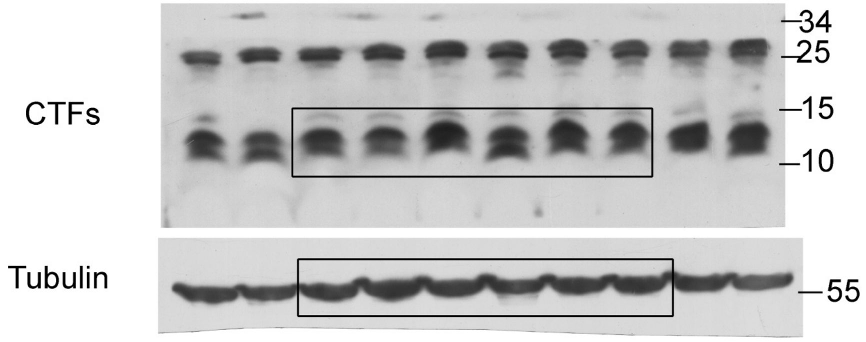

Supplementary Figure 15. Full scans of all blots in Supplementary Figure 2.

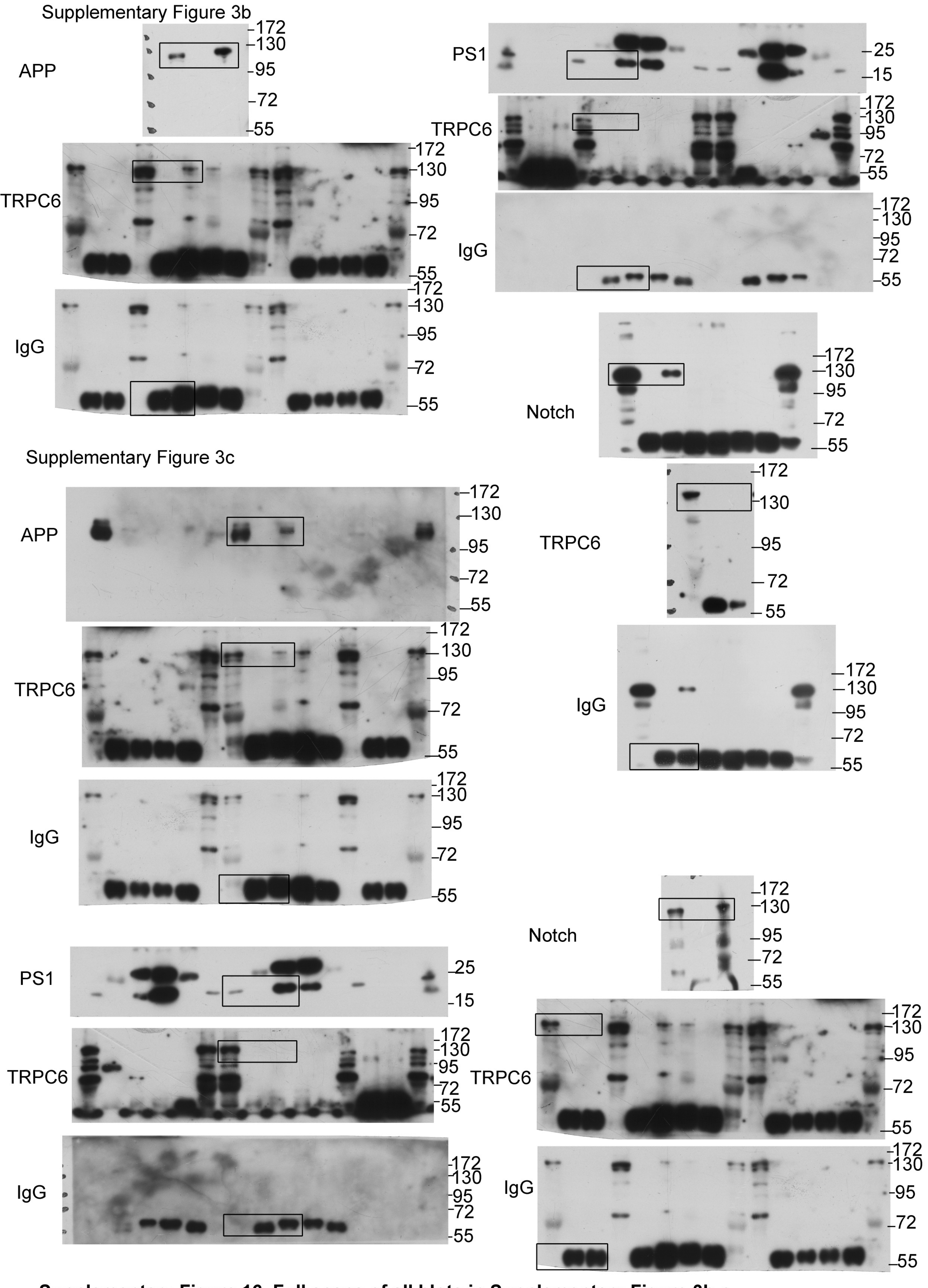

Supplementary Figure 16. Full scans of all blots in Supplementary Figure 3b-c.

Supplementary Figure 3d

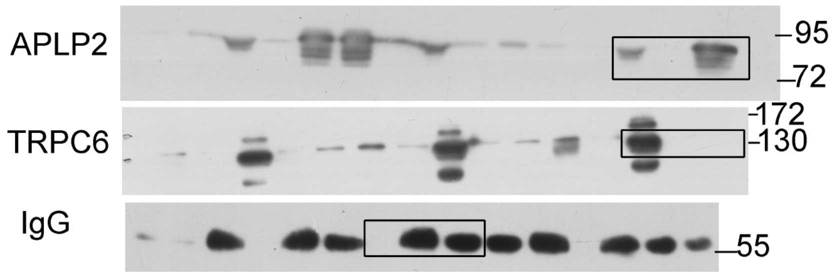

Supplementary Figure 3e

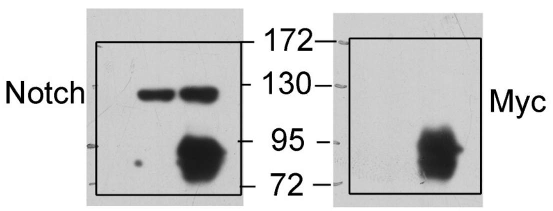

Supplementary Figure 3f

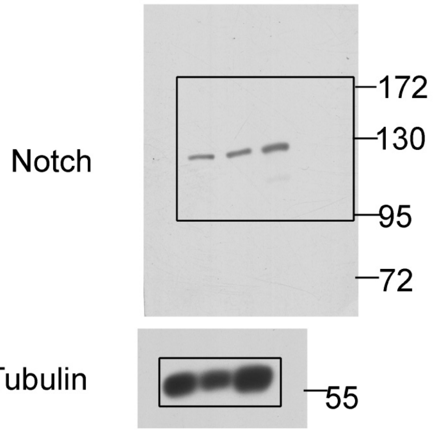

Supplementary Figure 4a

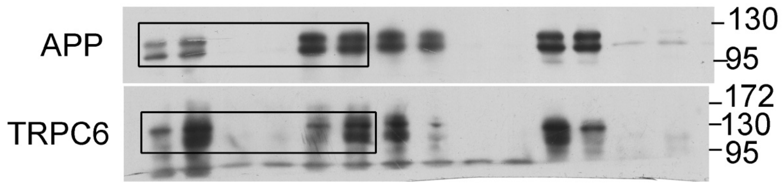

Supplementary Figure 4c

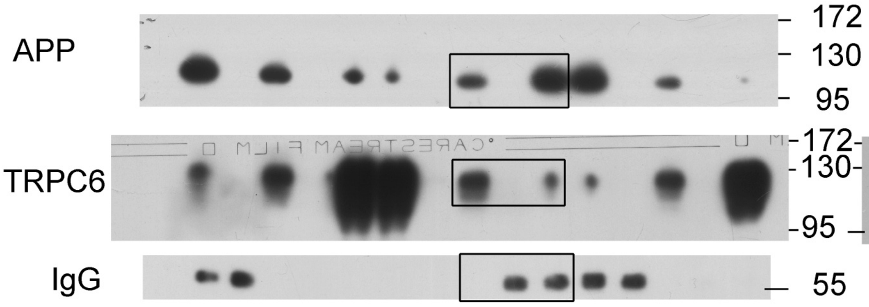

Supplementary Figure 4e

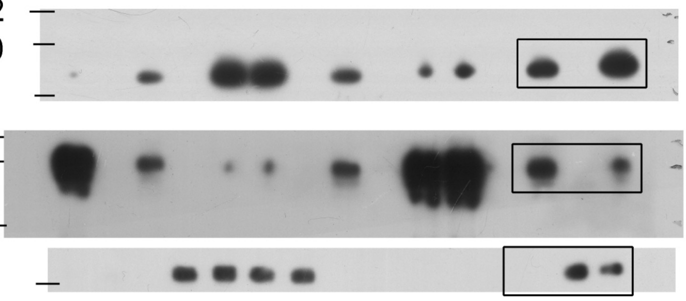

Supplementary Figure 4g

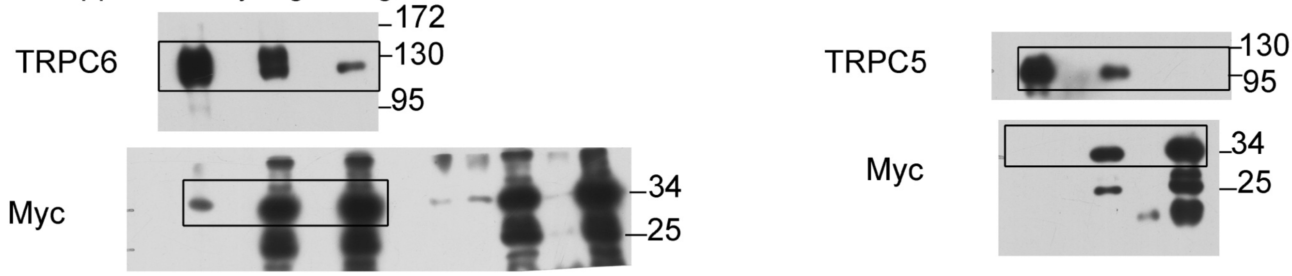

Supplementary Figure 17. Full scans of all blots in Supplementary Figure 3d-f and 4.

Supplementary Figure 7a

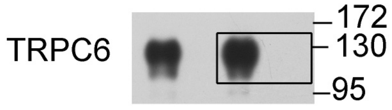

Supplementary Figure 7b

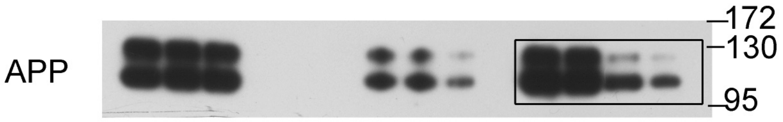

Supplementary Figure 7d

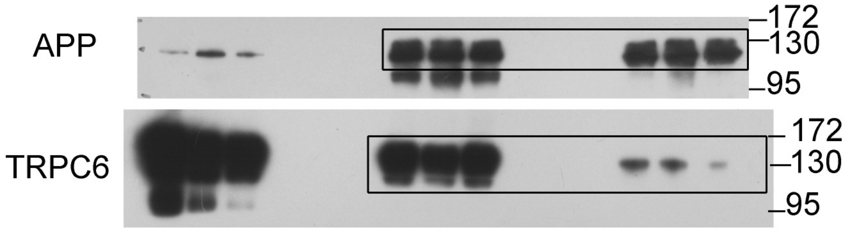

Supplementary Figure 7f

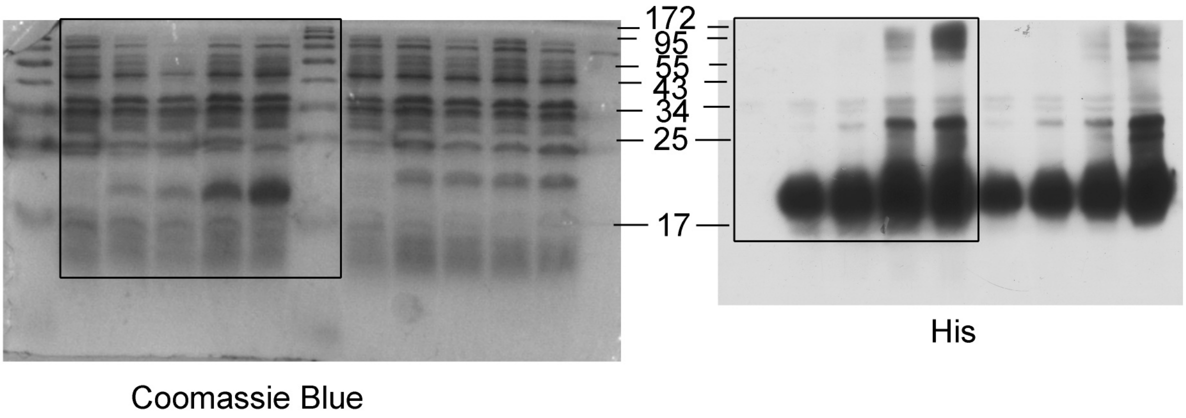

Supplementary Figure 7g

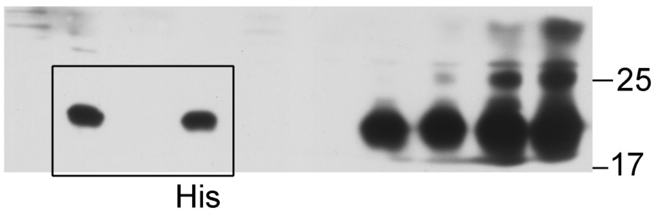

Supplementary Figure 18. Full scans of all blots in Supplementary Figure 7.
